# Evolutionary history and palaeoecology of brown bear in North-East Siberia re-examined using ancient DNA and stable isotopes from skeletal remains

**DOI:** 10.1038/s41598-019-40168-7

**Published:** 2019-03-14

**Authors:** Alba Rey-Iglesia, Ana García-Vázquez, Eve C. Treadaway, Johannes van der Plicht, Gennady F. Baryshnikov, Paul Szpak, Hervé Bocherens, Gennady G. Boeskorov, Eline D. Lorenzen

**Affiliations:** 1 Natural History Museum of Denmark, University of Copenhagen, DK-1350 Copenhagen K, Denmark; 2 Instituto de Xeoloxía Isidro Parga Pondal, ESCI, Campus de Elviña, Universidade da Coruña, 15071A Coruña, Spain; 3 Centre for Isotope Research, University of Groningen, Groningen, The Netherlands; 4 Zoological Institute, Russian Academy of Sciences, 199034 Saint Petersburg, Russia; 5 Department of Anthropology, Trent University, Peterborough, Ontario, K9L 0G2 Canada; 6 Department of Geosciences, Tübingen University, 72074 Tübingen, Germany; 7 Senckenberg Centre for Human Evolution and Palaeoenvironment, 72074 Tübingen, Germany; 8 Diamond and Precious Metals Geology Institute, Siberian Branch of Russian Academy of Sciences, 677980 Yakutsk, Russia

## Abstract

Over 60% of the modern distribution range of brown bears falls within Russia, yet palaeoecological data from the region remain scarce. Complete modern Russian brown bear mitogenomes are abundant in the published literature, yet examples of their ancient counterparts are absent. Similarly, there is only limited stable isotopic data of prehistoric brown bears from the region. We used ancient DNA and stable carbon (*δ*^13^C) and nitrogen (*δ*^15^N) isotopes retrieved from five Pleistocene Yakutian brown bears (one Middle Pleistocene and four Late Pleistocene), to elucidate the evolutionary history and palaeoecology of the species in the region. We were able to reconstruct the complete mitogenome of one of the Late Pleistocene specimens, but we were unable to assign it to any of the previously published brown bear mitogenome clades. A subsequent analysis of published mtDNA control region sequences, which included sequences of extinct clades from other geographic regions, assigned the ancient Yakutian bear to the extinct clade 3c; a clade previously identified from Late Quaternary specimens from Eastern Beringia and Northern Spain. Our analyses of stable isotopes showed relatively high *δ*^15^N values in the Pleistocene Yakutian brown bears, suggesting a more carnivorous diet than contemporary brown bears from Eastern Beringia.

## Introduction

The brown bear (*Ursus arctos*) has a broad Holarctic distribution, spanning Eurasia and North America^1^. Brown bears are also found on the islands of northeast Asia, including Hokkaido (Japan) and the southern Kuril Islands (Russia)^1^. Until the 20^th^ century, brown bears were common in most parts of Eurasia and in the western part of North America down to northern Mexico^2,3^. However, anthropogenic encroachment over the past century has resulted in reduced and fragmented brown bear populations^4^. Due to their extensive distribution, brown bears have become a model organism for studying biogeographic patterns across Eurasia and North America. Consequently, phylogeographic patterns of European brown bears were included in Hewitt’s seminal models of glacial contraction and postglacial expansion^5,6^.

**Figure 1:**
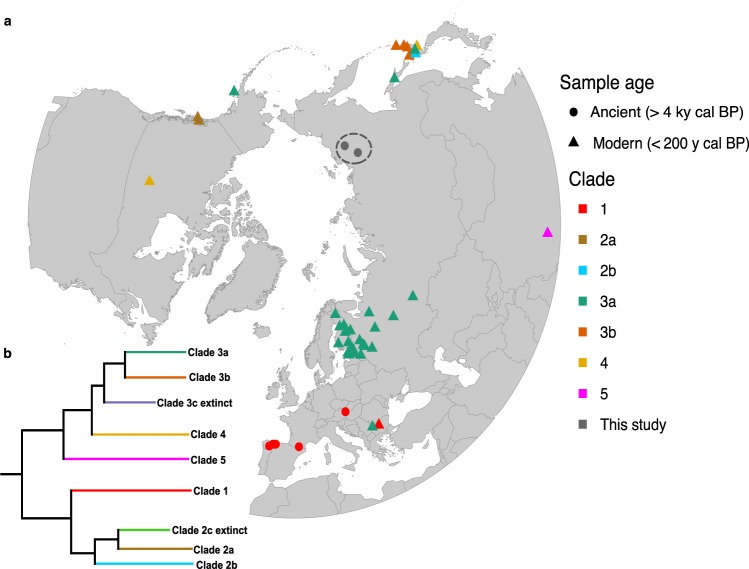
(**a**) Map showing the sample location of the brown bears analysed using mtDNA, including information on what clades they belong to; ancient samples (dots), modern samples (triangles). The five Yakutian specimens investigated using ancient DNA and stable isotopes (grey dots) are indicated by a dashed circle. Our mitogenome study included 129 sequences (published n = 128, this study n = 1). (**b**) Schematic phylogeny of brown bear mitogenome clades, adapted from^13^. Extinct clades 2c and 3c are not shown on the map.

Since the publication of the first brown bear phylogeographic study in 1994^7^, many studies have further characterized the phylogeny and phylogeography of the species using mitochondrial DNA (mtDNA)^8–12^. Based on patterns of variation in present-day brown bears, seven main extant clades have been described (Fig. 1). Clade 1 includes bears from western European populations^7^. Clade 2a is found only on the Admiralty, Baranof, and Chichagof (ABC) islands of Alaska. Clade 3a is the most widely distributed, and spans from Eastern Europe to western Alaska^1,13^. Clade 3b is found in both eastern Alaska and in brown bears from eastern Hokkaido (Japan), the Russian Far East, and southern Siberia. Clade 4 is most common amongst brown bears of North America, but has also been identified in Southern Hokkaido (Japan)^1,13^. Less widespread brown bear clades have been identified in Asia, including clade 5 in Tibet^1,14^. Other less widely distributed brown bear clades (not included in our analyses and therefore not shown in Fig. 1) have been found in North Africa, the Middle East^15,16^, and the Himalayas^17^. The mtDNA of extant polar bears (*Ursus maritimus*), clade 2b, is embedded within brown bears and is most closely related to clade 2a, the ABC brown bears^18^.

Fragments of the mitochondrial control region (CR) retrieved from ancient brown bear remains have been instrumental in developing our current understanding of the past biogeography of the species (e.g. refs.^10,12,19^). Ancient DNA analyses have documented shifting patterns of genetic diversity and structure, including the loss of genetic lineages during the Late Quaternary (such as clade 3c^10,19,20^). Such findings are indicative of more complex phylogeographic scenarios than initially suggested for the species by Taberlet and Bouvet^7^ (e.g. refs.^10,12,19,21^).

In this study, we extracted mitochondrial DNA of three Pleistocene subfossil brown bears collected in Yakutia, North-East Siberia, and assessed *δ*^13^C and *δ*^15^N of an additional two (totalling five) specimens. North-East Siberia is underrepresented in the large body of literature published on the species so far. Currently, there are no complete ancient mitochondrial sequences available in NCBI from the region, despite Russia covering >60% of the distribution range of brown bears^3^. Analysing our genetic data with the full panel of published brown bear mitogenomes, we reconstructed the maternal phylogeny of the species, to gain insight into the phylogenetic relationships among ancient Siberian brown bears and their modern contemporaries. To further elucidate the relationship between our Yakutian sequence and contemporary and extirpated brown bear populations, we included an analysis of the mtDNA CR, which included a larger temporal range of modern and ancient sequences.

Stable isotope data from Yakutian brown bears are limited^22^, compared with brown bears from other regions, or with other coeval large carnivores, limiting our understanding of their palaeoecology. We therefore compiled published isotopic data of Late Pleistocene megaherbivores and carnivores from North-East Siberia and Eastern Beringia, and compared them to the *δ*^13^C and *δ*^15^N isotopic compositions of our five Yakutian specimens.

## Results and Discussion

**Figure 2:**
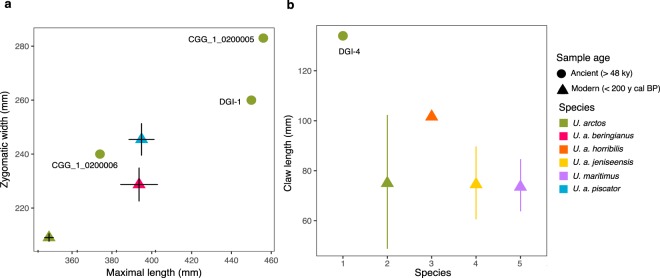
(**a**) Bivariate plot of the skull zygomatic width (in mm) and maximal length (in mm) of three of the ancient Yakutian bears from this study (green dots), and modern brown bears (*Ursus arctos* L.) from Siberia and the Russian Far East (triangles). Modern brown bears measurements are represented as mean ± SD (see Supplementary Table 1 for full details). (**b**) Plot representing the claw length along the outer curvature (in mm) of the ancient Yakutian bear (DGI-4) (green dot) and modern bears from Eurasia and North America (triangles). Lines represent an interval connecting minimal and maximal length values (see Supplementary Table 2).

We investigated five Pleistocene (one Middle Pleistocene and four Late Pleistocene) subfossil brown bears from Yakutia, one with an infinite ^14^C age (CGG_1_0200005) and the other four undated (CGG_1_0200006, CGG_1_0200007, DGI-1, and DGI-4). Morphological assessment of the individuals indicated that four of the five specimens, CGG_1_0200005, CGG_1_0200006, DGI-1, and DGI-4 were exceptionally large individuals (Fig. 2; Supplementary Tables 1–3).

### mtDNA assembly

Three of the individuals (CGG_1_0200005, CGG_1_0200006, and CGG_1_0200007) were subjected to DNA analysis. To reconstruct mitochondrial genomes, we isolated total DNA using ancient DNA methodologies and transformed it into sequencing libraries. One library of each of the three samples was pooled with other samples and shotgun sequenced on the Illumina HiSeq. 2500 platform. Based on mapping to the polar bear reference genome (GCF_000687225.1), the endogenous content of the three samples ranged from 0.06% to 10% (Table 1). The endogenous content of 10% in sample CGG_1_0200006 indicated good preservation, suggesting nuclear work on this Late Pleistocene specimen may be possible in future studies.

**Table 1:** Basic statistics derived from read processing and mapping.

Sample	Total reads	Nuclear mapping	Endogenous content	mtDNA mapping	Depth
CGG_1_0200005	7,322,879	20,897	0.29%	92	0.3x
CGG_1_0200006	164,297,287	16,563,819	10%	18,612	69x
CGG_1_0200007	7,097,255	4,282	0.06%	44	0.12x

Mapping of the reads to the brown bear mtDNA reference genome (NC_003427.1) varied substantially between specimens: CGG_1_0200005 yielded 92 unique reads; CGG_1_0200006 18,612 unique reads; and CGG_1_0200007 44 unique reads (Table 1). Based on the number of reads after filtering, we were able to assemble the complete mitogenome (defined as >90% of the genome, covered to at least 3x read depth) of CGG_1_0200006 at 69x. The assembled mitogenome was 17,022 bp, with some gaps in the repetitive region of the CR; a region that is difficult to assemble for ancient brown bear specimens, due to low coverage or ambiguous read alignment^23^. The low number of unique sequencing reads derived from CGG_1_0200005 and CGG_1_0200007 were not sufficient to assemble robust mitogenomes; thus, these two samples were not subjected to further DNA analysis. Ancient damage pattern estimates from mapDamage show that all our samples have terminal base substitutions, which authenticate the sequenced fragments as ancient (Supplementary Fig. 1).

### Ancient Yakutian mitogenome shifts clade 3 divergence estimate back > 25 ky

**Figure 3:**
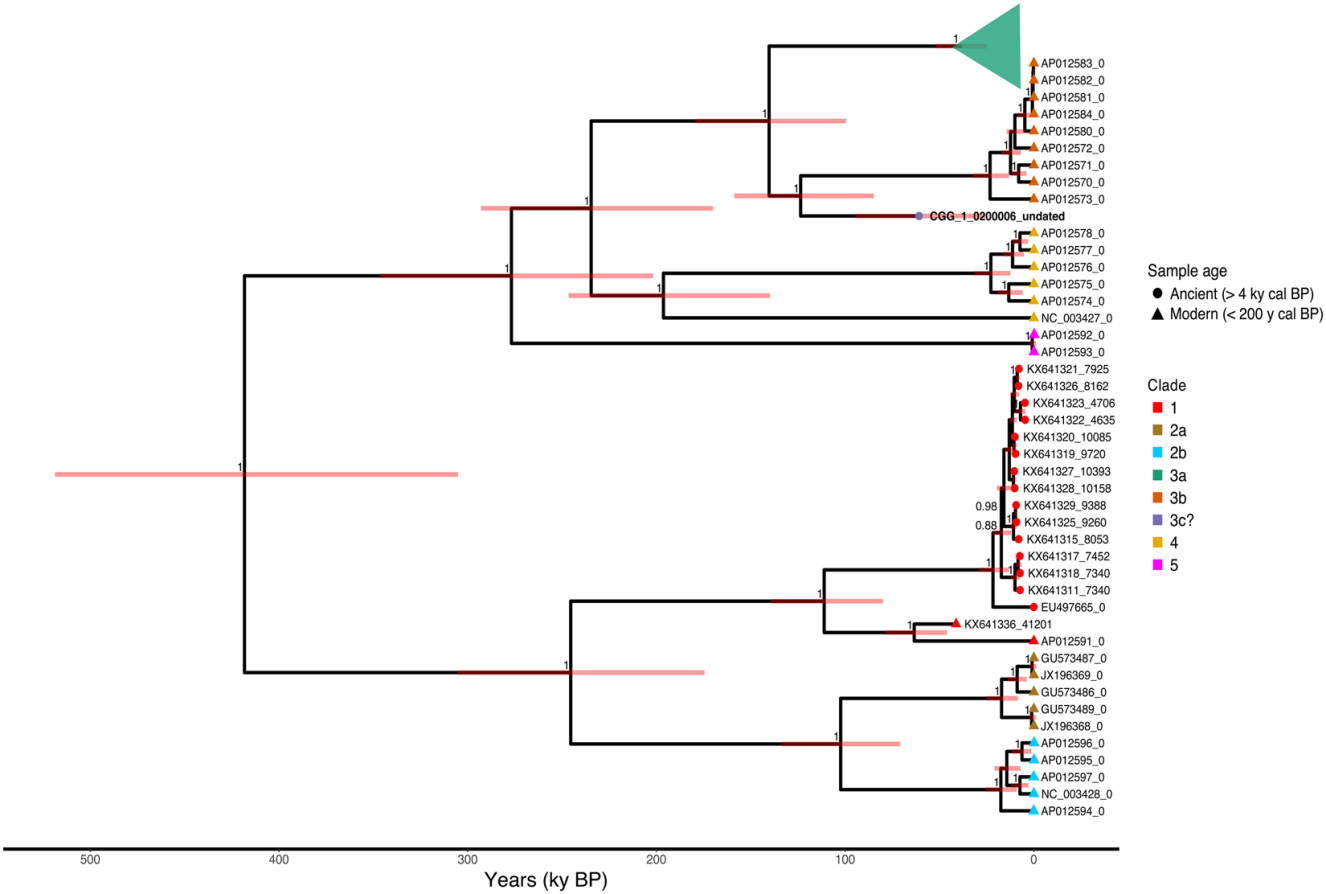
Tip-calibrated phylogeny of the brown bear mitogenomes analysed using BEAST. Branch labels indicate posterior clade probabilities >0.85. Tip label postfixes indicate sample age. Tips are coloured based on clade, and tip symbols indicate sample age. Nodes are centred on the median estimated divergence time, and red bars indicate the 95% HPD. Clade 3a was collapsed to facilitate visualization. Information on the specimens included in clade 3a can be found in Supplementary Table 3.

The mitogenome from CGG_1_0200006 was combined with 128 full mitochondrial genome sequences downloaded from NCBI: 17 ancient brown bears (41,201–4,635 years cal BP), 101 modern brown, and 10 polar bears; the mitochondrial lineage of the latter groups within the diversity of brown bears and hence was included in our analysis. MrBayes v3.2.6^24^ and BEAST v.1.8.2^25^ were used to estimate phylogenetic relationships. Both analyses yielded the same topology, and placed our Late Pleistocene Yakutian specimen as a sister lineage to clade 3b (as defined in Hirata *et al*. 2013) (Fig. 3; Supplementary Fig. 2). We ran BEAST v.1.8.2^25^ using a tip-dating approach to perform the molecular dating of CGG_1_0200006, which had not been radiocarbon dated, and to estimate clade divergence times. For CGG_1_0200006, this approach gave a mean age estimate of 61,826 years BP [HPD 95%: 27,866–96,783 years BP].

The lack of specimens representative of clade 3c in the mitogenome dataset (Fig. 3; Supplementary Fig. 2) meant that CGG_1_0200006 could not be assigned to a known clade, nor be confirmed as a novel lineage. To mitigate this, we generated a haplotype network of 225 mtDNA CR sequences representing clades 3a, 3b, 3c, and 4 from modern and ancient brown bears (Supplementary Table 4). We used PopART v.1.7^26^ to construct a median-joining network of the overlapping region of 270 bp of the CR, with clade 4 sequences as an outgroup.

**Figure 4:**
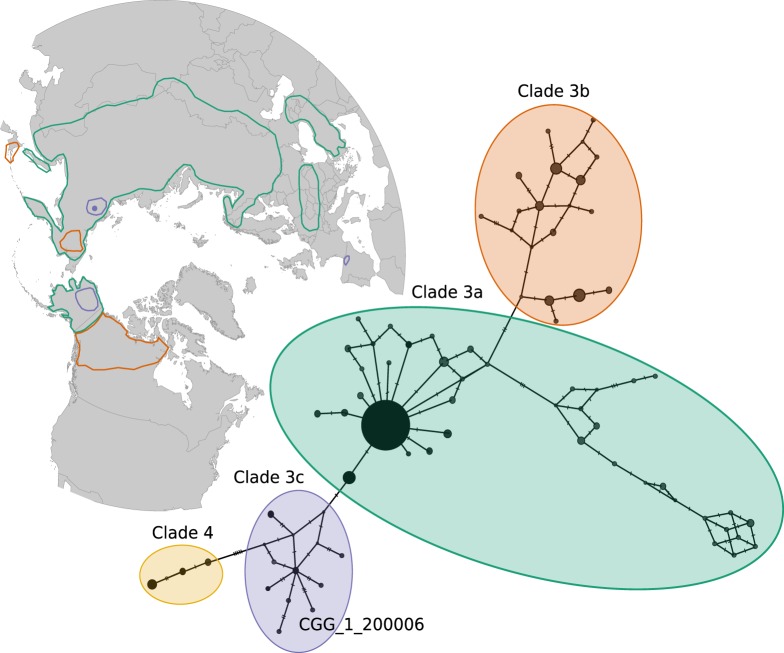
Clade 3 mtDNA CR haplotype network of 225 published sequences, indicating the location of the Yakutian specimen sequenced in this study (CGG_1_0200006). Specimen information listed in Supplementary Table 4. Circles are sized relative to haplotype frequency. Dashes indicate single-nucleotide mutations. The map represents the range of the clade 3 lineages (3a, 3b and 3c). Ranges were drawn based on^13^. Analysis was run with Clade 4 as an outgroup.

We find that CGG_1_0200006 grouped with extinct clade 3c brown bears (Fig. 4). Clade 3c has been identified in ancient Alaskan and Iberian brown bears dated >35 ky cal BP^10,19,20^. Our data provide further evidence that this clade was indeed widespread across the northern hemisphere during the Late Pleistocene.

We estimated a divergence time of clade 3c from clades 3a/b of 123 ky BP [HPD 95%: 90–164] ky BP. This predates previous estimates based on short fragments of mtDNA CR sequences by >25 ky^13,27^. Other studies of brown bear have estimated various time to the most recent common ancestor (tMRCA) for other clades, in particular influenced by the length of the alignment (single mtDNA genes versus whole mitogenomes) and the method of calibration (e.g. tip-dating, fossil calibration)^1,13,28^.

**Figure 5:**
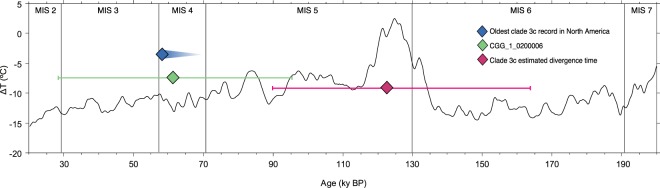
Curve of air temperature variation relative to the present (°C) through time^30^. Marine isotopic stadials (MIS) according to Lisiecki & Raymo^90^. Minimum age of the oldest record of a clade 3c brown bear of America (>59 ky BP, blue); Yakutian clade 3c bear (CGG_1_0200006) from this study (61,826 years BP [HPD 95%: 27,866–96,783 years BP], green); estimated clade 3c divergence time (123 ky BP [HPD 95%: 90–164] ky BP, pink).

Central Asia is accepted as the central point of origin and diversification of brown bears^2^, and colonization of other areas from this region would lead to the diversification of lineages. During the glacial periods of the Pleistocene, the decline in ocean levels created a land bridge between Eurasia and North America^29^. A previous study investigating several biogeographic scenarios for brown bear colonization of North America suggested the entrance of several brown bear clades into North America was initiated around 70 ky BP^13^. This is supported by our results, which imply that divergence of clade 3c took place at the beginning of Marine Isotope Stage 5 (MIS5), during an interglacial, where temperatures were higher than today^30^ (Fig. 5). Due to the contraction of the ice and improved climatic conditions, brown bears could have expanded across Asia and possibly Europe. The oldest radiocarbon date of clade 3c brown bears in Alaska is as infinite date of >59 ky BP^10^, and our divergence estimate supports that at the end of MIS 5 or the onset of MIS 4, when climates cooled, East Asian clade 3c brown bears may have crossed Beringia across the exposed Bering Land Bridge^13^. At present, only one extinct clade 3c brown bear individual has been recorded in Europe, so we can only speculate as to whether this reflects an as yet undiscovered clade 3c population, or vagrant bears that moved westwards^12^.

### Isotopic signatures differ in Eastern and Western Beringia

**Figure 6:**
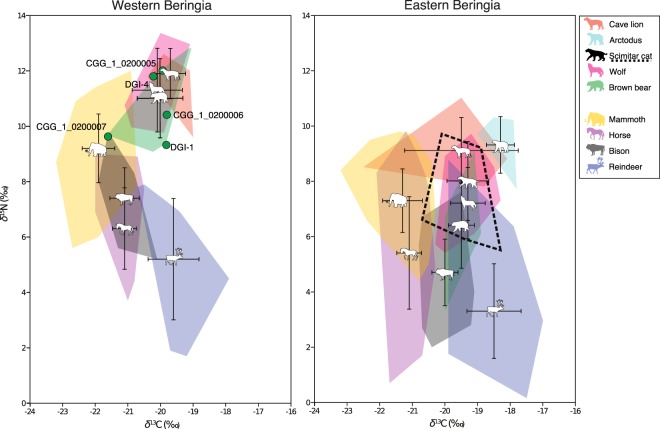
Bivariate plot of the stable carbon and nitrogen isotope values of the five Yakutian brown bears (green dots), and other pre-LGM megafauna from Western Beringia (left) and Eastern Beringia (right). Except the five bears analysed in this study, species are represented as mean ± 1 SD (see Supplementary Table 5 for full details) and the shaded areas represent the convex hulls^91^, which are defined as the minimum area polygon encompassing all points in bivariate space. Horse silhouette: Mercedes Yrayzoz (vectorized by T. Michael Keesey), license CC-BY-3.0 (https://creativecommons.org/licenses/by/3.0/). Scimitar cat and mammoth silhouettes: Sergiodlarosa (original images have been modified), license CC-BY-SA-3.0 (http://creativecommons.org/licenses/by-sa/3.0/).

The stable isotopic compositions (*δ*^13^C and *δ*^15^N) of the five Yakutian brown bears were measured and compared to other Beringian megafauna predating the Last Glacial Maximum (LGM; only samples >25 ky cal BP) (Fig. 6). At that time, the taxonomic composition of mammoth steppe mammalian communities was similar from Europe to Eastern Beringia^31^. Species included large predators such as felids (e.g. lions (*Panthera spelaea/Panthera atrox*) spanning Europe to Alaska and scimitar cats (*Homotherium serum*) in Eastern Beringia), bears (e.g. brown bears across the mammoth steppe and short-faced bears (*Arctodus simus*) in Eastern Beringia), and canids (e.g. wolves (*Canis lupus*) across the entire region and hyenas (*Crocuta crocuta*) in western Eurasia)^32^. Wide-ranging mammoth steppe megaherbivores included woolly mammoths (*Mammuthus primigenius*), reindeer (*Rangifer tarandus*), bison (*Bison sp*.), horses (*Equus ferus*), caprine bovids (*Bootherium cavifrons* in Beringia, and *Rupicapra rupicapra* in Europe), and Eurasian woolly rhinoceros (*Coelodonta antiquitatis*).

We compiled the isotopic signatures of various megafauna species from Western and Eastern Beringia, including the five Yakutian brown bears from this study (Fig. 6; Supplementary Table 5). Despite the potential age difference among the Western Beringia brown bear samples, our *δ*^13^C and *δ*^15^N analysis indicate that the carnivorous diet in brown bear from the region was sustained through time, despite climatic and environmental fluctuations^33^.

Our results also reflect differences between the isotopic signatures of megaherbivores of Western and Eastern Beringia (Fig. 6), in agreement with previous work^31,34^. Megaherbivores from Western Beringia have higher *δ*^15^N (difference from +1 to +2.7‰) and lower *δ*^13^C (difference from 0 to −1.1‰) compared to Eastern Beringia (See Supplementary Table 6). The general offset in all species likely reflects environmental differences between the two regions. Soil *δ*^15^N is a product of nitrogen-transforming microorganisms and is proportional to the total soil organic nitrogen, average annual temperatures and precipitation^35^; soil and plant *δ*^15^N are strongly correlated^36^ and herbivore *δ*^15^N reflects the nitrogen isotope composition of the plants consumed^37,38^. In Europe, regions with higher development of permafrost during the LGM yield the lowest collagen *δ*^15^N in large herbivores during the post-LGM period, around 23–14 kyr cal BP^39,40^. This is comparable to contemporary patterns in which *δ*^15^N in surface soils rise with increasing distance from a glacier front, due to an increase in soil development^41^. The glaciers in the Brooks and Alaska ranges—in the north and south of Eastern Beringia, respectively^42^-could be responsible for the low *δ*^15^N in this region relative to Western Beringia, where the distance to the glacial front in Western Eurasia was larger during and after the LGM^43^. In addition, other climatic conditions, such as less aridity in Alaska, have been suggested^33,44^.

### Isotopic signatures suggest Pleistocene brown bears from Western Beringia had carnivorous diets

Based on interspecific comparative palaeoecological analyses and paleoenvironmental data, we suggest that the high *δ*^15^N values in the Yakutian brown bears reflects a highly carnivorous diet. Pre-LGM clade 3c brown bears from Eastern Beringia^10^ had lower *δ*^15^N (presumably less carnivorous) compared to our Yakutian clade 3c brown bear (CGG_1_020000006), suggesting that carnivorous dietary habits were not linked to a mitochondrial haplogroup, but rather to palaeoecological factors.

In herbivores, interspecific differences in isotopic values have been linked to dietary specialization, and to interspecific differences in how food is digested^34,39,44–46^. Thus, differences exist among the hindgut fermenter horse relative to coeval ruminants, including bison. Reindeer has a diet strongly biased towards lichens that are ^13^C-enriched relative to vascular C_3_-plants^47^. Mammoth *δ*^15^N values are significantly higher than those of any other herbivore species^31^. In this way, despite the absolute differences in isotopic values of Western and Eastern Beringia (Fig. 6), the isotopic offset between megaherbivore species is preserved on both sides of Beringia.

This is not the case for wolves and brown bears, which implies that dissimilarities between these taxa are driven by dietary differences, rather than environmental variation impacting the entire food web. The difference between Western and Eastern Beringia for the brown bear is +4.6‰ for *δ*^15^N and −0.5‰ for *δ*^13^C, and in the case of the wolf is +4.1‰ for *δ*^15^N and −0.8‰ for *δ*^13^C (Supplementary Table 6). The other carnivore that is present on both sides is the lion, but the difference between regions is akin to the pattern seen in megaherbivores (+2.9‰ for *δ*^15^N and −0.2‰ for *δ*^13^C) (Supplementary Table 6). The overlap in the isotopic distribution among lions, wolves, and brown bears in Eastern Beringia is small, while in Western Beringia it is noticeable (Fig. 6). In Pleistocene Europe, wolves and brown bears competed with hyenas and lions; Pleistocene wolves exhibited lower *δ*^15^N values than contemporary cave hyenas and cave lions, despite their overlap with some cave lion individuals^48^. A similar scenario might have occurred in Eastern Beringia, but with more predators with which to compete, besides lions: scimitar cats and short-faced bears, which occupied a higher trophic position than wolves and brown bears. The reduced competition with other predators in Western Beringia may have provided brown bears and wolves with the opportunity to predate larger herbivores, rather than scavenging.

The typically high *δ*^15^N values in pre-LGM brown bears from North Western and Central Europe have also been attributed to competition with vegetarian cave bears (*Ursus spelaeus)*^32,49,50^. Cave bears have been reported in Western Beringia^51,52^, but not in Eastern Beringia. In Western Beringia, such competition may have contributed to a more carnivorous diet in brown bears. This may in turn have enabled exceptionally large body sizes, as in four of our specimens; body size in extant brown bears is correlated with meat availability^53^. Such a large size would have been advantageous in competition with the other large predators of the Western Beringia mammoth steppe^32^.

## Conclusions

This study presents the first combined ancient DNA and stable isotope (*δ*^13^C and *δ*^15^N) investigation of Pleistocene brown bears from Yakutia. Using shotgun sequencing, we generated a complete mitogenome sequence (69x) that is distinct from any previously published brown bear mitogenome clades. Phylogenetic comparisons to extant and extinct brown bear sequences assigned this individual to the extinct clade 3c, which has previously been found in Eastern Beringia and Spain. Thus, this mitochondrial genome represents the first available full mtDNA for clade 3c. This mitogenome allowed us to generate a new estimation of the tMRCA of clade 3c to the other clade 3 bears, predating previous estimates by >25 ky and supporting the diversification of this clade in Western Beringia, followed by the colonization of North America across the Bering Land Bridge. Stable isotope analyses show high *δ*^15^N values in pre-LGM brown bears from Yakutia, indicating that these bears were more carnivorous than brown bears in Eastern Beringia. This could reflect reduced competition among carnivores in the region, enabling brown bears to predate larger herbivores, and possibly also competition with vegetarian cave bears present in Western Beringia, but absent in Eastern Beringia.

Importantly, our study raises a phylogenetic database issue. The lack of complete mitogenomes for some clades might bias phylogenetic inferences. Thus, if possible, we suggest performing complementary analyses using single genes/partial sequences (e.g. partial control region, cytochrome b) largely available through NBCI, whenever there is a reduced representation of age, geography, and/or lineages at the full mitogenome level. Also, to further assess evolutionary processes and to test phylogeographic hypotheses, extended ancient DNA studies—in particular from Pleistocene specimens—derived from underrepresented geographic areas are needed. Moreover, investigations that combine ancient DNA and isotopic analyses may lead to a more complete understanding of the evolutionary ecology of phylogeographic changes in Pleistocene terrestrial large mammals, and we suggest they be performed more systematically.

## Materials and Methods

### Samples

Five subfossil bear samples were excavated during various paleontological surveys in Yakutia from 2005 through 2016 (see Fig. 1 and Supplementary Tables 1, 2 for precise locations) as incomplete skeletal remains. For three samples, some cranial material was recovered and, for the remaining two specimens, an upper canine fragment and a claw were recovered.

The largest brown bear skull from the Uyandina River, CGG_1_0200005, belonged to an adult male, and was radiocarbon dated at the University of Groningen (see section below). Morphological measurements of this skull exceed the corresponding parameters not only for other ancient and modern bear skulls from Yakutia, but also the maximal sizes of the largest representatives of modern brown bear subspecies from Eurasia (*U. a. beringianus*, Amur River region, Russian Far East and *U. a. piscator*, Kamtchatka Peninsula)^54^ (Supplementary Table 1).

The second skull, CGG_1_0200006, was of smaller size and morphological identification assigned it to an adult female (Supplementary Table 1). The degree of preservation of the skulls, as well as the bone remains of accompanying fauna, suggest that this taphocenosis could have been formed during the MIS 3 (ca. 60–27 kyr cal BP) the time of the greatest warming during second half of the Late Pleistocene, when special conditions were created for the death and burial of mammoth fauna.

The third specimen, CGG_1_0200007, represents a fragment of brown bear skull, cerebral cortex, that could not be used to assess the size of this individual (Supplementary Table 1). The fragment, that was dark-brown in colour and strongly fossilized, was found at the Ulakhan Sullar outcrop, Adycha River, Verkhoyansk district^55^.

The tooth sample (DGI-1) from the Deputatsky village that belonged to a large brown bear, was stratigraphically assigned to the Late Pleistocene. Unfortunately, it was not possible to perform direct measurements of the skull of this bear (Supplementary Table 1).

Finally, measurements of the claw sample (DGI-4) found in 2016 at the Novosibirsk Islands Archipelago (the extreme north of Yakutia), indicate that it was a very large specimen compared to modern brown bear claws from Yakutia and other regions, as well as modern polar bear claws (Supplementary Information; Supplementary Table 2). Based on stratigraphy, this bear sample was assigned to the Upper Pleistocene.

### Radiocarbon dating and stable isotope data

The lower jaw bone of CGG_1_0200005 was radiocarbon dated at the University of Groningen, based on an acid-based collagen extracted fraction^56^. The collagen was ^14^C dated by Accelerator Mass Spectrometry^57^, yielding an infinite age for the ^14^C method: >45 ky BP (GrA-66592). This is expressed in conventional ^14^C years, and corresponds to an age >48,000 years^58^. CGG_1_0200005 and CGG_1_0200006 were excavated from the same geological layer, thus CGG_1_0200006 is assumed to be from the same period as CGG_1_0200005, although this specimen was not radiocarbon dated. CGG_1_0200007 was not radiocarbon dated either, but initial superficial collagen-based assessment at the Russian Academy of Sciences (on the extent of skeletal fossilisation, as per^59^) indicated a likely temporal origin for this sample in the late Middle Pleistocene (ca. ~120 to 200 ky BP).

For specimens CGG_1_0200005, CGG_1_0200006, and CGG_1_0200007, collagen was extracted from powdered bone samples by demineralizing with 0.5 M HCl at room temperature for 4 h under constant motion. Samples were then rinsed to neutrality and treated with 0.1 M NaOH at room temperature for successive 20 min treatments until no colour change was observed in the solution. Samples were rinsed to neutrality, solubilized in 10^−3^ M HCl at 75 °C for 36 h, and then the solution containing the collagen was transferred to a glass vial and lyophilized. Stable carbon and nitrogen isotopic compositions were measured at Trent University, using a Nu Horizon (Nu Instruments, Wrexham, UK) continuous-flow isotope ratio mass spectrometer coupled to a EuroEA 3000 (Eurovector, Milan, Italy) elemental analyser. Analytical uncertainty was determined to be ±0.20‰ for both *δ*^13^C and *δ*^15^N^60^. Analysed samples were only included in plots and interpretations if they were characterized by acceptable atomic C:N ratios (2.9–3.6) and minimums for wt% C, wt% N, and wt% collagen^61^. The ^14^C dated jaw bone also produced stable isotope ratios. These were very similar to those obtained from the skulls.

Further stable isotope analyses were conducted in Tübingen, where the collagen was extracted for all five brown bear specimens, with a similar protocol^62^. For the claw sample (DGI-4), a pretreatment with 2:1 chloroform:methanol was applied to remove possible fat contaminants^63^. The stable carbon and nitrogen isotopic compositions were performed in duplicate at the Institute of Environmental Science and Technology (ICTA, Barcelona) using a Thermo Flash 1112 (Thermo Scientific VC) elemental analyzer coupled to a Thermo Delta V Advantage mass spectrometer with a Conflo III interface. This measures the ratios of ^13^C/^12^C and ^15^N/^14^N relative to a standard (V-PDB for carbon and AIR for nitrogen). The international laboratory standard, IAEA 600 (caffeine), was used. Analytical uncertainty was determined to be ±0.20‰ for both *δ*^13^C and *δ*^15^N, based on multiple measurements of collagen extracted from modern bones of camel (*Camelus dromedarius*) and elk (*Alces alces*). The isotopic values of the tooth sample (DGI-1) were converted into equivalent *δ*^13^C and *δ*^15^N of collagen (*δ*^13^Ccoll and *δ*^15^Ncoll) taking into consideration the mean differences observed between collagen and bone in brown bear (*δ*^13^Cbone = *δ*^13^Cdentine − 0.67; *δ*^15^Nbone = *δ*^15^Ndentine − 1.47)^32^. The *δ*^13^C value of the claw sample (DGI-4) was transformed into equivalent *δ*^13^C values of collagen (*δ*^13^Ccoll) considering the mean enrichment of +2‰ observed between collagen and keratin^64^.

In order to contextualize the isotopic data of the five brown bears from this study, 426 additional isotopic records from Eastern and Western Beringia were compiled from the literature^10,22,34,44,65–69^. All data is pre-LGM and have a radiocarbon or stratigraphic dating. The dataset consisted of megaherbivores that were present on both sides of the Bering Strait: mammoth (*Mammuthus primigenius*), horse (*Equus sp*.), bison (*Bison sp*.), and reindeer (*Rangifer tarandus*), in addition to all available carnivores: brown bear, wolf (*Canis sp*.), cave lion (*Panthera spelaea*), scimitar cat (*Homotherium serum*), and short-faced bear (*Arctodus simus*). See Supplementary Table 5. Bivariate analyses for *δ*^13^C and *δ*^15^N were conducted in Past3 v3.20^70^.

### Ancient DNA extraction and amplification

We drilled 50–70 mg of bone powder from each specimen and extracted DNA using a modified version of the protocol from^71^: samples were incubated overnight with the extraction buffer at 42 °C instead of at 37 °C, the bone powder was pelleted out of suspension, and the supernatant concentrated down to 150–200 μl for each sample using 30 kDa Amicon centrifugal filter unit (Millipore). Binding buffer was added 13x to the concentrated supernatant and DNA was purified with MinElute columns (Qiagen), following the manufacturer’s instructions with the exception of a 15-minute incubation at 37 °C during the elution step. DNA extracts were transformed into sequencing libraries in 25 μl reactions following the protocol from^72^ with the following modifications: the initial DNA fragmentation was not performed; MinElute kit (Qiagen) was used for the purification steps. DNA libraries were indexed using KAPA HiFi uracil + premix (KAPA Biosystems). The number of cycles for index PCRs was determined from qPCR analysis. The resulting libraries were quantified on an Agilent 2100 Bioanalyser, pooled at equimolar concentration and sequenced on an Illumina HiSeq 2500 SR 80 basepairs (bp).

### Data processing

Sequencing reads processing (adapter trimming with AdapterRemoval v2.2.2^73^), mapping (read alignment, PCR duplicate removal, and indel realignment), and damage analyses (mapDamage v2.0.6^74^) were performed using the PALEOMIX v1.2.13.1 pipeline^75^. BWA-aln v0.7.15^76^ with disabled seed was used for mapping, following the authors’ recommendation. Reads shorter than 30 bp were discarded during adaptor trimming. Reads were mapped to the polar bear nuclear reference to estimate the endogenous content of our libraries (GCF_000687225.1^77^), and to the mitochondrial brown bear reference (NC_003427.1^78^). GATK v4.0.4.0^79^ was used to perform indel realignment within the PALEOMIX v1.2.13.1 pipeline^74^. ANGSD v0.919^80^ was used to generate mitochondrial genome consensus sequences from the unique reads BAM files. Base calling was performed under highest effective depth (EBD) at each site, with EBD being the product of the mapping quality and scores for the base under consideration^81^.

### Phylogenetic and network analysis

We included consensus sequences covering >90% of the brown bear mitochondrial reference genome (NC_003427.1^66^) in the phylogenetic analysis. Consensus sequences were combined with 128 published mitochondrial genomes of ancient brown bears (n = 17, spanning from 41,201 to 4,635 years cal BP), modern brown (n = 101), and polar bears (n = 10) using MAFFT v7.392^82^ (see Supplementary Table 3). A repetitive region of the control region was excluded from the alignment due to ambiguous alignment and missing data in the NCBI sequences. The final size of the alignment (including transfer RNA –tRNAs-, ribosomal RNA –rRNAs-, protein coding-genes, and partial control region) was 15,928 bp. PartitionFinder v1.1.1^83^ was used to determine the partition scheme and substitution model for the mitogenome alignment using the Bayesian Information Criterion and the greedy algorithm. PartitionFinder v1.1.1^83^ suggested three partitions for our data: rRNAs and tRNAs; 1^st^ and 2^nd^ codon protein-coding genes; 3^rd^ codon protein-codon genes; and partial control region. The best models suggested by the Bayesian Information Criterion are HKY + I model for the rRNAs and tRNAs; HKY + I for the 1^st^ and 2^nd^ codon partition; the HKY + G for the 3^rd^ codon partition; and HKY + G + I for the partial control region.

We constructed a Bayesian phylogeny using MrBayes v3.2.6^24^ with two runs and four chains of 1 × 10^7^ Markov chain Monte Carlo (MCMC) generations and sampling every 1 × 10^3^ generations with a 25% burn-in. Convergence was assessed with Tracer v1.6^84^. Trees were summarized with the majority-rule consensus approach, using posterior probability as a measure of clade support.

We also constructed a phylogeny in BEAST v.1.8.2^25^ using the ancient dated sequences to estimate a mutation rate across the mitochondrial genome and divergence dates of the lineages. BEAST was also used to estimate the age of CGG_1_0200006. The alignment used in the BEAST analysis was a reduced version of the alignment analysed with MrBayes, as we did not include a published ancient undated sample (see Supplementary Table 3). Phylogenetic age estimation was performed using a tip-dating method^85^. The prior posterior distribution of the tip date of the undated sample was set between zero (present day) and one million years, following the analyses of reference^22^. Substitution rate priors for all the partitions were assigned an independent mutation rate prior according to^86^. A cross-validation method was used to estimate the date of samples with known age^23^. A date-randomization test was performed to test for the temporal signal in the data. TipDatingBeast v1.0–8^87^ was used to generate 20 date-randomized BEAST xml files and compare the parameter estimates of the real dataset with the date-randomized datasets. Our date-randomization test was passed, indicating that there is a temporal signal in our dataset (Supplementary Fig. 3).

All BEAST analyses were run in two independent MCMC chains of 1 × 10^8^ generations each, sampling trees and model parameters every 1 × 10^3^ generations. Tracer v1.6^84^ was used to combine and inspect the results of each run and to determine the convergence of each parameter, all of which had ESS values >200. We identified the Maximum Clade Credibility (MCC) tree in TreeAnnotator v1.8.0, and visualized and graphically edited the MCC tree using ggtree^88^.

To further evaluate the phylogenetic placement of the brown bear mtDNA generated in this study, we compiled a dataset of 225 published control region (CR) sequences for clades 3a, 3b, 3c and 4 (Supplementary Table 4). The consensus sequence for CGG_1_0200006 was aligned to the sequences in the CR dataset using MAFFT v7.392^82^. The alignment was trimmed down to 270 bp, which allowed for the incorporation of sequences from the extinct clade 3c^10^. A haplotype network was generated using the median-joining algorithm^89^ implemented in the program PopART v.1.7^26^.

### Accession number

Complete mitogenome for the specimen CGG_1_0200006 is stored in the NCBI GenBank with accession number MH255807.

## Supplementary information

Supplementary Information
