# Supplementary material for: Evolutionary history and palaeoecology of brown bear in North-East Siberia re-examined using ancient DNA and stable isotopes from skeletal remains
Source: Sci Rep. 2019 Mar 14;9:4462. doi: 10.1038/s41598-019-40168-7 (PMC6418263; doi:10.1038/s41598-019-40168-7)
Supplement: Supplementary file 1 — Supplementary Information [file 41598_2019_40168_MOESM1_ESM.docx]

**Evolutionary history and palaeoecology of brown bear in North-East Siberia re-examined using ancient DNA and stable isotopes from skeletal remains**

Alba Rey-Iglesia^1^, Ana García-Vázquez^2^, Eve C. Treadaway^1^, Johannes van der Plicht^3^, Gennady F. Baryshnikov^4^, Paul Szpak^5^, Hervé Bocherens^6,7^, Gennady G. Boeskorov^8^ & Eline D. Lorenzen^1*^

^1^Natural History Museum of Denmark, University of Copenhagen, DK-1350 Copenhagen K, Denmark.

^2^Instituto de Xeoloxía Isidro Parga Pondal, ESCI, Campus de Elviña, Universidade da Coruña, 15071 A Coruña, Spain.

^3^Centre for Isotope Research, University of Groningen, the Netherlands

^4^Zoological Institute, Russian Academy of Sciences, 199034 Saint Petersburg, Russia.

^5^Department of Anthropology, Trent University, Peterborough, Ontario, Canada K9L 0G2

^6^Department of Geosciences, Tübingen University, 72074 Tübingen, Germany

^7^Senckenberg Centre for Human Evolution and Palaeoenvironment, 72074 Tübingen, Germany

^8^Diamond and Precious Metals Geology Institute, Siberian Branch of Russian Academy of Sciences, 677980 Yakutsk, Russia.

**Supplementary Information**

**Data collection and description of the brown bear claw (DGI-4):**

In the summer of 2016, on the Bolshoy Lyakhovsky Island in the Laptev Sea (approximal coordinates N73º48ʺ, E141º09ʺ) (the Novosibirsk Islands Archipelago, the extreme north of Yakutia), a very large claw (DGI-4) of a predatory beast was found. It has a rounded shape and a dark brown color, like modern brown bears. However, at present there are no brown bears on this island, only polar bears are found there. The polar bear has completely different claws, they are black, relatively small, sharp and slightly curved^92^. Bear fossil claw was found in the frozen sediments of the Upper Pleistocene together with the bones of fossil mammals of the Mammoth fauna (mammoth, steppe bison). In the Late Pleistocene, the Bolshoy Lyakhovsky Island was at times connected with the mainland coast and at this time brown bears sometimes penetrated there^55^. Judging by the curved shape and size, the claw originated from the middle finger of the brown bear's front paw. On this finger, bears usually have the longest claw. The size of this claw is extremely large. Its length is much longer than the claws of the modern brown bear from Yakutia and even exceeds the length of the claws of the largest subspecies of the brown bear from Eurasia (Supplementary Table 2). The length of the claw from the Bolshoi Lyakhovsky Island is also greater than the length of the claws of the very large grizzly bear *Ursus arctos horribilis*, whose claws are longer than that of the big brown bear Ursus arctos middendorffii^93^. Thus, the claw from the Bolshoy Lyakhovsky Island belonged to a brown bear of exceptionally large size.

**Supplementary Figures:**

Supplementary Figure 1. Deamination patterns obtained using mapDamage at the ends of the sequencing reads. Left panel: C to T deamination rate at the 5’ end. Right panel: G to A rate at the 3’ end. Colours correspond to the three sequenced specimens.


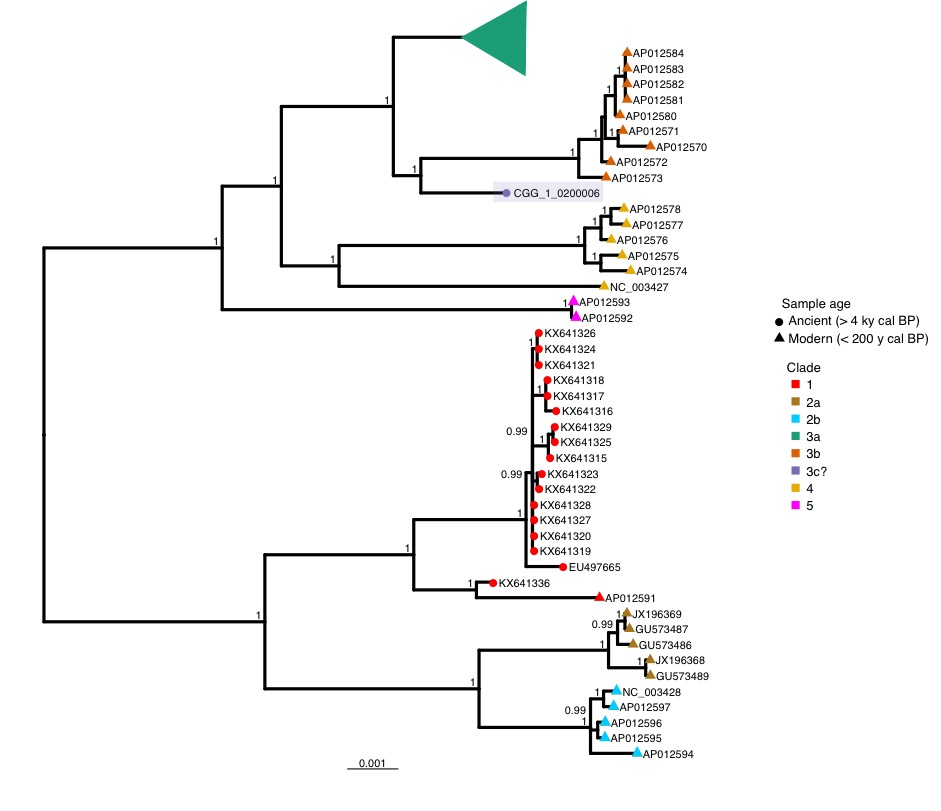


Supplementary Figure 2. MrBayes phylogeny of the 129 brown bear mitogenomes analysed, showing the location of CGG_1_0200006, highlighted in purple. Specimens are the same as represented in the map in Fig. 1. Tips are coloured based on clade affiliation, and tip symbols indicate whether samples are ancient or modern. Only posterior probabilities > 0.9 are shown. Clade 3a was collapsed to facilitate visualization. Information on the specimens included in clade 3a can be found in Supplementary Table 1. The scale represents expected changes per site.

Supplementary Figure 3. Comparison of the substitution rates estimated for the different mitogenome partitions using BEAST using both the real data and 20 date-randomized datasets from the date-randomization test (DRT).

Supplementary Table 1. Dimensional characteristics of the skulls of fossil and modern brown bears (*Ursus arctos* L.) in Siberia and the Russian Far East

| Measurements, mm | Fossil, Pleistocene, north of Yakutia (East Siberia) | | | | Modern, adult males (Boeskorov & Baryshnikov, 2013) | | |
| --- | --- | --- | --- | --- | --- | --- | --- |
|  | Vicinity of the Deputatsky village, appr. N68°57', E 138°24', Late Pleistocene | Indigirka river basin, Uyandina river, N68.33.759, E.144.45.980,  Late Pleistocene, more 45.000 BP | Indigirka river basin, Uyandina river, N68.33.759, E.144.45.980, Late Pleistocene, probably has same geological age as DGI-2 | Verkhoyansk district, Adycha R., N67.694436, E135.737638, Middle (?) Pleistocene | Medium sized morph | Largest subspecies in Eurasia | |
|  | Fragment of the upper canine | Fragments of skull and mandible | Fragment of bulla timpani | Piece of skull | *U. arctos* *ssp.*, Yakutia, n=66 | *U. arctos beringianus*, Amur River basin, n=8 | *U. arctos piscator*, Kamtchatka Peninsula, n=9 |
|  | Lab Code DGI-1 | Lab Code CGG_1_0200005 (DGI-2) GrA-65241 | Lab Code CGG_1_0200006 (DGI-3) | Lab Code CGG_1_0200007 (DGI-5) | l i m i t  X±m | l i m i t  X±m | l i m i t  X±m |
|  | Adult male, giant | Adult male, giant | Large, but not giant, probably female | Medium sized |  |  |  |
| Maximal length | About 450 | 456 | 374 | - | 315 – 392  348.42±2.20 | 356 – 432  393.63±9.25 | 359 – 420  394.89±6.33 |
| Zygomatic  width | About 260 | 283 | About 240 | - | 185 – 231  209.05±1.43 | 206 – 246.6  228.71±6.17 | 226 – 265  245.44±5.90 |
| Maximum height of the skull | - | 157 | - | 132 | 115 – 144  130.94±0.87 | 113.6 – 150  130.61±5.49 | 120 – 151  134.0±3.83 |

Notes. The specimen from the vicinity of the Deputatsky village is very large, but unfortunately there is no possibility to measure the skull directly.

Supplementary Table 2. The length of the middle claw (III finger) on the front paw of bears (in mm).

| Species, subspecies, geographical region, geological age | Brown bear *Ursus arctos* | | | | Polar bear  *Ursus maritimus*, modern |
| --- | --- | --- | --- | --- | --- |
|  | fossil | modern | | | modern |
|  | *Ursus arctos sp.,* Bolshoy Lyakhovsky Island, Late Pleistocene (?),  Lab Code DGI-4 | *Ursus arctos jeniseensis*, Yakutia, n=16 | *Ursus arctos*, Eurasia  (Geptner et al., 1967) | *Ursus arctos horribilis*, Alaska, Canada  (Burt and Grossenheider, 1956) | *Ursus maritimus*, Russian Arctic (Geptner et al., 1967) |
|  |  | Limit  X±m |  |  |  |
| Claw length along the outer curvature | 134 | 62 – 87  68.5±1.71 | 50 - 100 | About 4 inches (about 101.6 mm) | Up to 65 – 70;  Up to 82* |

* our own data

**References:**

1. Geptner, V. G. *et al*. Mammals of the Soviet Union. Familia Ursidae. *Vysshaya Skhola* **2** (1) [in Russian] (1967, Moscow).
2. Burt, W. H. & Grossenheider, R. P. A Field Guide to the Mammals. Houghton Mifflin (Company Boston, 1956).

Supplementary Table 3. Detailed information on the specimens used in this study to estimate phylogenetic relationships using mitogemic data.

| **GenBank code** | **Latitude** | **Longitude** | **Age group** | **14C Age** | **Location** | **Reference** | **Notes** |
| --- | --- | --- | --- | --- | --- | --- | --- |
| CGG_1_0200005 | 68.56265 | 144.7663 | Ancient | >48kybp | Indigirka basin  (Yakutia) | This study |  |
| CGG_1_0200006 | 68.56265 | 144.7663 | Ancient | >48kybp | Indigirka basin  (Yakutia) | This study |  |
| CGG_1_0200007 | 67.694436 | 135.7376 | Ancient | >48kybp | Verkhoyansk  (Yakutia) | This study |  |
| EU497665.1 | 42.779369 | 1.300805 | Ancient | Unknown | Guzet Shelter  (France) | Bon et al. 2008 |  |
| KX641315.1 | 42.615833 | -7.11917 | Ancient | 8,053 | Pena Paleira Cave,  Folgoso do Courel (Spain) | Fortes et al. 2016 |  |
| KX641316.1 | 42.615833 | -7.11917 | Ancient | 7,340 | Pena Paleira Cave,  Folgoso do Courel (Spain) | Fortes et al. 2016 |  |
| KX641317.1 | 42.615833 | -7.11917 | Ancient | 7,452 | Pena Paleira Cave,  Folgoso do Courel (Spain) | Fortes et al. 2016 |  |
| KX641318.1 | 42.615833 | -7.11917 | Ancient | 7,340 | Spain, PenaPaleira, FolgosoDoCourelCave | Fortes et al. 2016 |  |
| KX641319.1 | 43.05 | -6.12222 | Ancient | 9,720 | Sima de los Osos de Somiedo Cave, Somiedo (Spain) | Fortes et al. 2016 |  |
| KX641320.1 | 43.05 | -6.12222 | Ancient | 10,085 | Sima de los Osos de Somiedo Cave, Somiedo (Spain) | Fortes et al. 2016 |  |
| KX641321.1 | 43.05 | -6.12222 | Ancient | 7,925 | Sima de los Osos de Somiedo Cave, Somiedo (Spain) | Fortes et al. 2016 |  |
| KX641322.1 | 43.05 | -6.12222 | Ancient | 4,635 | Sima de los Osos de Somiedo Cave, Somiedo (Spain) | Fortes et al. 2016 |  |
| KX641323.1 | 43.05 | -6.12222 | Ancient | 4,706 | Sima de los Osos de Somiedo Cave, Somiedo (Spain) | Fortes et al. 2016 |  |
| KX641324.1 | 43.05 | -6.12222 | Ancient | Undated* | Sima de los Osos de Somiedo Cave, Somiedo (Spain) | Fortes et al. 2016 |  |
| KX641325.1 | 43.051667 | -5.9575 | Ancient | 9,260 | Pozu La Cigacha Cave  Teverga (Spain) | Fortes et al. 2016 |  |
| KX641326.1 | 43.051667 | -5.9575 | Ancient | 8,162 | Pozu La Cigacha Cave,  Teverga (Spain) | Fortes et al. 2016 |  |
| KX641327.1 | 43.055 | -5.95306 | Ancient | 10,393 | Pozu LaVeiga Retuertu Cave,  Quirós (Spain) | Fortes et al. 2016 |  |
| KX641328.1 | 43.099722 | -5.33 | Ancient | 10,158 | La Canal Fuerte Cave,  Campocaso (Spain) | Fortes et al. 2016 |  |
| KX641329.1 | 43.099722 | -5.33 | Ancient | 9,388 | La Canal Fuerte Cave,  Campocaso (Spain) | Fortes et al. 2016 |  |
| KX641336.1 | 47.95298 | 16.75343 | Ancient | 41,201 | Winden AmSee, WindenBärenhöhle (Austria) | Fortes et al. 2016 |  |
| AP012559.1 | 43.403 | 142.657 | Modern |  | Central Hokkaido  (Japan) | Hirata et al. 2013 | not exact coordinates |
| AP012560.1 | 43.403 | 142.657 | Modern |  | Central Hokkaido  (Japan) | Hirata et al. 2013 | not exact coordinates |
| AP012561.1 | 43.403 | 142.657 | Modern |  | Central Hokkaido  (Japan) | Hirata et al. 2013 | not exact coordinates |
| AP012562.1 | 43.403 | 142.657 | Modern |  | Central Hokkaido  (Japan) | Hirata et al. 2013 | not exact coordinates |
| AP012563.1 | 43.403 | 142.657 | Modern |  | Central Hokkaido  (Japan) | Hirata et al. 2013 | not exact coordinates |
| AP012564.1 | 43.403 | 142.657 | Modern |  | Central Hokkaido  (Japan) | Hirata et al. 2013 | not exact coordinates |
| AP012565.1 | 43.403 | 142.657 | Modern |  | Central Hokkaido  (Japan) | Hirata et al. 2013 | not exact coordinates |
| AP012566.1 | 43.403 | 142.657 | Modern |  | Central Hokkaido  (Japan) | Hirata et al. 2013 | not exact coordinates |
| AP012567.1 | 43.403 | 142.657 | Modern |  | Central Hokkaido  (Japan) | Hirata et al. 2013 | not exact coordinates |
| AP012568.1 | 43.403 | 142.657 | Modern |  | Central Hokkaido  (Japan) | Hirata et al. 2013 | not exact coordinates |
| AP012569.1 | 43.403 | 142.657 | Modern |  | Central Hokkaido  (Japan) | Hirata et al. 2013 | not exact coordinates |
| AP012570.1 | 44 | 144.8216 | Modern |  | Eastern Hokkaido  (Japan) | Hirata et al. 2013 | not exact coordinates |
| AP012571.1 | 44 | 144.8216 | Modern |  | Eastern Hokkaido  (Japan) | Hirata et al. 2013 | not exact coordinates |
| AP012572.1 | 44 | 144.8216 | Modern |  | Eastern Hokkaido  (Japan) | Hirata et al. 2013 | not exact coordinates |
| AP012573 | 44 | 144.8216 | Modern |  | Eastern Hokkaido  (Japan) | Hirata et al. 2013 | not exact coordinates |
| AP012574 | 42.583408 | 142.7836 | Modern |  | Southern Hokkaido  (Japan) | Hirata et al. 2013 | not exact coordinates |
| AP012575 | 42.583408 | 142.7836 | Modern |  | Southern Hokkaido  (Japan) | Hirata et al. 2013 | not exact coordinates |
| AP012576 | 42.583408 | 142.7836 | Modern |  | Southern Hokkaido  (Japan) | Hirata et al. 2013 | not exact coordinates |
| AP012577 | 42.583408 | 142.7836 | Modern |  | Southern Hokkaido  (Japan) | Hirata et al. 2013 | not exact coordinates |
| AP012578 | 42.583408 | 142.7836 | Modern |  | Southern Hokkaido  (Japan) | Hirata et al. 2013 | not exact coordinates |
| AP012579 | 50.485807 | 142.826157 | Modern |  | Sakhalin  (Japan) | Hirata et al. 2013 | not exact coordinates |
| AP012580.1 | 44.145223 | 145.867682 | Modern |  | Kunashiri Island  (Japan) | Hirata et al. 2013 | not exact coordinates |
| AP012581.1 | 45.067932 | 147.788229 | Modern |  | Etorofu Island  (Japan) | Hirata et al. 2013 | not exact coordinates |
| AP012582.1 | 45.067932 | 147.788229 | Modern |  | Etorofu Island  (Japan) | Hirata et al. 2013 | not exact coordinates |
| AP012583.1 | 45.067932 | 147.788229 | Modern |  | Etorofu Island  (Japan) | Hirata et al. 2013 | not exact coordinates |
| AP012584.1 | 45.067932 | 147.788229 | Modern |  | Etorofu Island  (Japan) | Hirata et al. 2013 | not exact coordinates |
| AP012585 | 57.267824 | 61.038738 | Modern |  | Ekaterinburg  (Ural Mountains) | Hirata et al. 2013 | not exact coordinates |
| AP012586 | 57.267824 | 61.038738 | Modern |  | Ekaterinburg  (Ural Mountains) | Hirata et al. 2013 | not exact coordinates |
| AP012587 | 57.267824 | 61.038738 | Modern |  | Ekaterinburg  (Ural Mountains) | Hirata et al. 2013 | not exact coordinates |
| AP012588 | 58.708083 | 32.183958 | Modern |  | Novgorod | Hirata et al. 2013 | not exact coordinates |
| AP012589 | 58.708083 | 32.183958 | Modern |  | Novgorod | Hirata et al. 2013 | not exact coordinates |
| AP012590.1 | 42.884154 | 24.773287 | Modern |  | Balkan Mountains  (Bulgaria) | Hirata et al. 2013 | not exact coordinates |
| AP012591.1 | 42.884154 | 24.773287 | Modern |  | Balkan Mountains  (Bulgaria) | Hirata et al. 2013 | not exact coordinates |
| AP012592.1 | 32.450275 | 88.138539 | Modern |  | Tibet | Hirata et al. 2013 | not exact coordinates |
| AP012593.1 | 32.450275 | 88.138539 | Modern |  | Tibet | Hirata et al. 2013 | not exact coordinates |
| AP012594.1 | 43.768619 | 142.479769 | Modern |  | Asahikawa Municipal Asahiyama Zoo, Japan | Hirata et al. 2013 | not exact coordinates |
| AP012595.1 | 43.768619 | 142.479769 | Modern |  | Asahikawa Municipal Asahiyama Zoo, Japan | Hirata et al. 2013 | not exact coordinates |
| AP012596.1 | 43.768619 | 142.479769 | Modern |  | Asahikawa Municipal Asahiyama Zoo, Japan | Hirata et al. 2013 | not exact coordinates |
| AP012597.1 | 43.768619 | 142.479769 | Modern |  | Asahikawa Municipal Asahiyama Zoo, Japan | Hirata et al. 2013 | not exact coordinates |
| GU573486.1 | 57.685293 | -134.489 | Modern |  | Admiralty Island  (Alaska) | Lindqvist et al. 2010 |  |
| GU573487.1 | 57.685293 | -134.489 | Modern |  | Admiralty Island  (Alaska) | Lindqvist et al. 2010 |  |
| GU573489.1 | 56.997371 | -135 | Modern |  | Baranof Island  (Alaska) | Lindqvist et al. 2010 |  |
| GU573491.1 | 57.504942 | -153.369 | Modern |  | Kodiak Island  (Alaska) | Lindqvist et al. 2010 |  |
| HQ685901.1 | 59.245 | 26.391 | Modern |  | Lääne-Virumaa and Ida-Virumaa,  (Estonia) | Keis et al. 2013 |  |
| HQ685902.1 | 58.918 | 25.68 | Modern |  | Järvamaa, Jgevamaa, Raplamaa and Viljansimaa (Estonia) | Keis et al. 2013 |  |
| HQ685903.1 | 58.918 | 25.68 | Modern |  | Järvamaa  (Estonia) | Keis et al. 2013 |  |
| HQ685904.1 | 58.388 | 26.792 | Modern |  | Järvamaa and Tartumaa  (Estonia) | Keis et al. 2013 |  |
| HQ685905.1 | 58.918 | 25.68 | Modern |  | Järvamaa  (Estonia) | Keis et al. 2013 |  |
| HQ685906.1 | 58.467 | 24.695 | Modern |  | Pärnumaa and Lääne-Virumaa  (Estonia) | Keis et al. 2013 |  |
| HQ685907.1 | 58.918 | 25.68 | Modern |  | Järvamaa  (Estonia) | Keis et al. 2013 |  |
| HQ685908.1 | 59.245 | 26.391 | Modern |  | Lääne-Virumaa  (Estonia) | Keis et al. 2013 |  |
| HQ685909.1 | 58.072 | 27.167 | Modern |  | Põlvamaa, Tartumaa and Viljandimaa (Estonia) | Keis et al. 2013 |  |
| HQ685910.1 | 59.181 | 27.34 | Modern |  | Ida-Virumaa  (Estonia) | Keis et al. 2013 |  |
| HQ685911.1 | 58.918 | 25.68 | Modern |  | Estonia, Järvamaa | Keis et al. 2013 |  |
| HQ685912.1 | 59.245 | 26.391 | Modern |  | Estonia, Lääne-Virumaa and J,gevamaa and Tartimaa | Keis et al. 2013 |  |
| HQ685913.1 | 58.072 | 27.167 | Modern |  | Estonia, Põlvamaa | Keis et al. 2013 |  |
| HQ685914.1 | 58.927 | 24.728 | Modern |  | Estonia, Raplamaa | Keis et al. 2013 |  |
| HQ685915.1 | 59.245 | 26.391 | Modern |  | Lääne-Virumaa | Keis et al. 2013 |  |
| HQ685916.1 | 59.897 | 31.729 | Modern |  | Russia, Leningrad-oblast | Keis et al. 2013 |  |
| HQ685917.1 | 59.897 | 31.729 | Modern |  | Russia, Leningrad-oblast | Keis et al. 2013 |  |
| HQ685918.1 | 59.897 | 31.729 | Modern |  | Russia, Leningrad-oblast | Keis et al. 2013 |  |
| HQ685919.1 | 59.897 | 31.729 | Modern |  | Russia, Leningrad-oblast | Keis et al. 2013 |  |
| HQ685920.1 | 59.897 | 31.729 | Modern |  | Russia, Leningrad-oblast | Keis et al. 2013 |  |
| HQ685921.1 | 59.897 | 31.729 | Modern |  | Russia, Leningrad-oblast | Keis et al. 2013 |  |
| HQ685922.1 | 59.897 | 31.729 | Modern |  | Russia, Leningrad-oblast | Keis et al. 2013 |  |
| HQ685923.1 | 59.897 | 31.729 | Modern |  | Russia, Leningrad-oblast | Keis et al. 2013 |  |
| HQ685924.1 | 59.897 | 31.729 | Modern |  | Russia, Leningrad-oblast | Keis et al. 2013 |  |
| HQ685925.1 | 59.897 | 31.729 | Modern |  | Russia, Leningrad-oblast | Keis et al. 2013 |  |
| HQ685926.1 | 59.897 | 31.729 | Modern |  | Russia, Leningrad-oblast | Keis et al. 2013 |  |
| HQ685927.1 | 59.947 | 40.58 | Modern |  | Russia, Vologda-oblast | Keis et al. 2013 |  |
| HQ685928.1 | 63.277 | 42.953 | Modern |  | Russia, Arhangelsk-oblast | Keis et al. 2013 |  |
| HQ685929.1 | 63.277 | 42.953 | Modern |  | Russia, Arhangelsk-oblast | Keis et al. 2013 |  |
| HQ685930.1 | 63.277 | 42.953 | Modern |  | Russia, Arhangelsk-oblast | Keis et al. 2013 |  |
| HQ685931.1 | 63.277 | 42.953 | Modern |  | Russia, Arhangelsk-oblast | Keis et al. 2013 |  |
| HQ685932.1 | 63.277 | 42.953 | Modern |  | Russia, Arhangelsk-oblast | Keis et al. 2013 |  |
| HQ685933.1 | 63.277 | 42.953 | Modern |  | Russia, Arhangelsk-oblast | Keis et al. 2013 |  |
| HQ685934.1 | 63.277 | 42.953 | Modern |  | Russia, Arhangelsk-oblast | Keis et al. 2013 |  |
| HQ685935.1 | 63.277 | 42.953 | Modern |  | Russia, Arhangelsk-oblast | Keis et al. 2013 |  |
| HQ685936.1 | 63.277 | 42.953 | Modern |  | Russia, Arhangelsk-oblast | Keis et al. 2013 |  |
| HQ685937.1 | 58.605 | 49.812 | Modern |  | Russia, Kirov-oblast | Keis et al. 2013 |  |
| HQ685938.1 | 58.605 | 49.812 | Modern |  | Russia, Kirov-oblast | Keis et al. 2013 |  |
| HQ685939.1 | 58.605 | 49.812 | Modern |  | Russia, Kirov-oblast | Keis et al. 2013 |  |
| HQ685940.1 | 58.605 | 49.812 | Modern |  | Russia, Kirov-oblast | Keis et al. 2013 |  |
| HQ685941.1 | 58.605 | 49.812 | Modern |  | Russia, Kirov-oblast | Keis et al. 2013 |  |
| HQ685942.1 | 58.605 | 49.812 | Modern |  | Russia, Kirov-oblast | Keis et al. 2013 |  |
| HQ685943.1 | 58.605 | 49.812 | Modern |  | Russia, Kirov-oblast | Keis et al. 2013 |  |
| HQ685944.1 | 58.605 | 49.812 | Modern |  | Russia, Kirov-oblast | Keis et al. 2013 |  |
| HQ685945.1 | 58.605 | 49.812 | Modern |  | Russia, Kirov-oblast | Keis et al. 2013 |  |
| HQ685946.1 | 58.605 | 49.812 | Modern |  | Russia, Kirov-oblast | Keis et al. 2013 |  |
| HQ685947.1 | 58.268 | 32.442 | Modern |  | Russia, Novgorod-oblast | Keis et al. 2013 |  |
| HQ685948.1 | 58.268 | 32.442 | Modern |  | Russia, Novgorod-oblast | Keis et al. 2013 |  |
| HQ685949.1 | 58.268 | 32.442 | Modern |  | Russia, Novgorod-oblast | Keis et al. 2013 |  |
| HQ685950.1 | 57.041 | 34.685 | Modern |  | Russia, Tver-oblast | Keis et al. 2013 |  |
| HQ685951.1 | 57.041 | 34.685 | Modern |  | Russia, Tver-oblast | Keis et al. 2013 |  |
| HQ685952.1 | 57.041 | 34.685 | Modern |  | Russia, Tver-oblast | Keis et al. 2013 |  |
| HQ685953.1 | 57.041 | 34.685 | Modern |  | Russia, Tver-oblast | Keis et al. 2013 |  |
| HQ685954.1 | 57.169 | 29.269 | Modern |  | Russia, Pskov-oblast | Keis et al. 2013 |  |
| HQ685955.1 | 64.526 | 28.318 | Modern |  | Finland, Kainuu | Keis et al. 2013 |  |
| HQ685956.1 | 60.846 | 26.775 | Modern |  | Finland, Kymi and Satakunta | Keis et al. 2013 |  |
| HQ685957.1 | 64.526 | 28.318 | Modern |  | Finland, Kainuu and Central-Finland | Keis et al. 2013 |  |
| HQ685958.1 | 60.846 | 26.775 | Modern |  | Finland, Kymi | Keis et al. 2013 |  |
| HQ685959.1 | 61.558 | 22.125 | Modern |  | Finland, Satakunta | Keis et al. 2013 |  |
| HQ685960.1 | 67.598 | 26.669 | Modern |  | Finland, Lappi | Keis et al. 2013 |  |
| HQ685961.1 | 63.114 | 27.385 | Modern |  | Finland, North-Savo and Kymi | Keis et al. 2013 |  |
| HQ685962.1 | 64.753 | 25.993 | Modern |  | Finland, Oulu | Keis et al. 2013 |  |
| HQ685963.1 | 64.526 | 28.318 | Modern |  | Finland, Kainuu | Keis et al. 2013 |  |
| HQ685964.1 | 62.803 | 30.148 | Modern |  | Finland, North-Karjala | Keis et al. 2013 |  |
| JX196368.1 | 56.997371 | -135 | Modern |  | NorthAmerica, Alaska, BaranofIsland, Sitka | Miller et al. 2012 |  |
| JX196369.1 | 57.685293 | -134.489 | Modern |  | NorthAmerica, Alaska, AdmiraltyIsland | Miller et al. 2012 |  |
| NC03427.1 | 54 | -105 | Modern |  | North America | Delisle & Strobeck 2002 | not exact coordinates |
| NC03428.1 | 54 | -105 | Modern |  | North America | Delisle & Strobeck 2002 | not exact coordinates |

*This sample was not dated when we conducted the analysis. However, it has subsequently been dated to 7,120 ± 30 BP (7,958 ± 18 y cal BP) (García et al. 2017).

References:

1. Bon, C. *et al*. Deciphering the complete mitochondrial genome and phylogeny of the extinct cave bear in the Paleolithic painted cave of Chauvet. *Proc. Natl. Acad. Sci. USA* **105**, 17447–17452 (2008).
2. Fortes, G.G. et al. (2016). Ancient DNA reveals differences in behaviour and sociality between brown bears and extinct cave bears. *Mol. Ecol*. **25**, 4907–4918 (2016).
3. Hirata, D. et al. Molecular phylogeography of the brown bear (*Ursus arctos*) in Northeastern Asia based on analyses of complete mitochondrial DNA sequences. *Mol. Biol. Evol.* **30**, 1644–1652 (2013).
4. Lindqvist, C. et al. Complete mitochondrial genome of a Pleistocene jawbone unveils the origin of polar bear. *Proc. Natl. Acad. Sci. U.S.A.* **107**, 5053-5057 (2010).
5. Keis, M. et al. Complete mitochondrial genomes and a novel spatial genetic method reveal cryptic phylogeographical structure and migration patterns among brown bears in north‐western Eurasia. *J. Biogeogr.* **40**, 915-927 (2013).
6. Miller, W. et al. Polar and brown bear genomes reveal ancient admixture and demographic footprints of past climate change. *Proc. Natl. Acad. Sci. U.S.A.* ***109***, 2382-2390 (2012).
7. Delisle, I. & Strobeck, C. Conserved Primers for Rapid Sequencing of the Complete Mitochondrial Genome from Carnivores, Applied to Three Species of Bears. *Mol. Biol. Evol.* **19**, 357–361 (2002).

Supplementary Table 4. Detailed information on the specimens used to perform mtDNA control region haplotype networks

| **Clade** | **NCBI_code** | **Cave/Geographical origin** | **Radiocardon dating (calBP)** | **Reference** | **Base pairs** |
| --- | --- | --- | --- | --- | --- |
| 3a | KF182305 | Niedźwiedzia (Poland) | Pleistocene | Baca et al. 2013 | 269 |
| 3b | AY082847-AY082811 | Fairbanks | Ancient | Barnes et al. 2002 | 271 |
| 3b | AY082861-AY082825 | Fairbanks area | 22883 ± 305 | Barnes et al. 2002 | 271 |
| 3b | AY082857-AY082821 | Lower Goldstream | 18243 ± 237 | Barnes et al. 2002 | 271 |
| 3b | AY082855-AY082819 | Gold Hill | 16929 ± 157 | Barnes et al. 2002 | 271 |
| 3b | AY082864-AY082828 | Lower Goldstream | 18444 ± 301 | Barnes et al. 2002 | 271 |
| 3b | AY082846-AY082810 | Lower Goldstream | 24005 ± 344 | Barnes et al. 2002 | 271 |
| 3b | AY082863-AY082827 | Goldstream | 14697 ± 336 | Barnes et al. 2002 | 271 |
| 3b | AY082862-AY082826 | Goldstream | - | Barnes et al. 2002 | 271 |
| 3b | AY082854-AY082818 | Gold Hill | - | Barnes et al. 2002 | 271 |
| 3b | AY082860-AY082824 | Cripple Creek | 14486 ± 362 | Barnes et al. 2002 | 271 |
| 3b | AY082849-AY082813 | Goldstream | 17493 ± 259 | Barnes et al. 2002 | 271 |
| 3c | AY082868-AY082832 | Cripple Creek | 40805 ± 1051 | Barnes et al. 2002 | 271 |
| 3c | AY082866-AY082830 | Cripple Creek | 50800±1900 (sin calibrar) | Barnes et al. 2002 | 271 |
| 3b | AY082852-AY082816 | Ester Creek | 11535 ± 169 | Barnes et al. 2002 | 271 |
| 3b | AY082858-AY082822 | Lower Goldstream | 14460 ± 342 | Barnes et al. 2002 | 271 |
| 3b | AY082856-AY082820 | Rosie Creek | 18151 ± 278 | Barnes et al. 2002 | 271 |
| 3c | AY082872-AY082836 | Goldstream | 45228 ± 704 | Barnes et al. 2002 | 271 |
| 3c | AY082870-AY082834 | Cripple Creek | >56900 | Barnes et al. 2002 | 271 |
| 3c | AY082867-AY082831 | Cripple Creek | >53900 | Barnes et al. 2002 | 271 |
| 3b | AY082848-AY082812 | Engineer Creek | 13907 ± 225 | Barnes et al. 2002 | 271 |
| 3b | AY082851-AY082815 | Ester Creek | 17391 ± 250 | Barnes et al. 2002 | 271 |
| 3b | AY082853-AY082817 | Engineer Creek | 10895 ± 154 | Barnes et al. 2002 | 271 |
| 3b | AY082850-AY082814 | Goldstream | 16360 ± 417 | Barnes et al. 2002 | 271 |
| 3c | AY082873-AY082837 | Cripple Creek | 46186 ± 1318 | Barnes et al. 2002 | 271 |
| 3c | AY082869-AY082833 | Cripple Creek | 51684 ± 4255 | Barnes et al. 2002 | 271 |
| 3b | AY082859-AY082823 | Gold Hill | 19039 ± 227 | Barnes et al. 2002 | 271 |
| 3b | AY082865-AY082829 | Banks near Fox | 24808 ± 236 | Barnes et al. 2002 | 271 |
| 3c | AY082871-AY082835 | Fairbanks Creek | >59000 | Barnes et al. 2002 | 271 |
| 3a | AP012559.1 | Central Hokkaido | Modern | Hirata et al. 2013 | 16962 |
| 3a | AP012560.1 | Central Hokkaido | Modern | Hirata et al. 2013 | 16962 |
| 3a | AP012561.1 | Central Hokkaido | Modern | Hirata et al. 2013 | 16962 |
| 3a | AP012562.1 | Central Hokkaido | Modern | Hirata et al. 2013 | 16962 |
| 3a | AP012563.1 | Central Hokkaido | Modern | Hirata et al. 2013 | 16962 |
| 3a | AP012564.1 | Central Hokkaido | Modern | Hirata et al. 2013 | 16962 |
| 3a | AP012565.1 | Central Hokkaido | Modern | Hirata et al. 2013 | 16962 |
| 3a | AP012566.1 | Central Hokkaido | Modern | Hirata et al. 2013 | 16962 |
| 3a | AP012567.1 | Central Hokkaido | Modern | Hirata et al. 2013 | 16962 |
| 3a | AP012568.1 | Central Hokkaido | Modern | Hirata et al. 2013 | 16962 |
| 3a | AP012569.1 | Central Hokkaido | Modern | Hirata et al. 2013 | 16962 |
| 3b | AP012570.1 | Eastern Hokkaido | Modern | Hirata et al. 2013 | 16962 |
| 3b | AP012571.1 | Eastern Hokkaido | Modern | Hirata et al. 2013 | 16962 |
| 3b | AP012572.1 | Eastern Hokkaido | Modern | Hirata et al. 2013 | 16962 |
| 3b | AP012573 | Eastern Hokkaido | Modern | Hirata et al. 2013 | 16962 |
| 4 | AP012574 | Southern Hokkaido | Modern | Hirata et al. 2013 | 16962 |
| 4 | AP012575 | Southern Hokkaido | Modern | Hirata et al. 2013 | 16962 |
| 4 | AP012576 | Southern Hokkaido | Modern | Hirata et al. 2013 | 16962 |
| 4 | AP012577 | Southern Hokkaido | Modern | Hirata et al. 2013 | 16962 |
| 4 | AP012578 | Southern Hokkaido | Modern | Hirata et al. 2013 | 16962 |
| 3a | AP012579 | Sakhalin, Japan | Modern | Hirata et al. 2013 | 16962 |
| 3b | AP012580.1 | Kunashiri Island, Japan | Modern | Hirata et al. 2013 | 16962 |
| 3b | AP012581.1 | Etorofu Island | Modern | Hirata et al. 2013 | 16962 |
| 3b | AP012582.1 | Etorofu Island | Modern | Hirata et al. 2013 | 16962 |
| 3b | AP012583.1 | Etorofu Island | Modern | Hirata et al. 2013 | 16962 |
| 3b | AP012584.1 | Etorofu Island | Modern | Hirata et al. 2013 | 16962 |
| 3a | AP012585 | Ural Mountains, Ekaterinburg | Modern | Hirata et al. 2013 | 16962 |
| 3a | AP012586 | Ural Mountains, Ekaterinburg | Modern | Hirata et al. 2013 | 16962 |
| 3a | AP012587 | Ural Mountains, Ekaterinburg | Modern | Hirata et al. 2013 | 16962 |
| 3a | AP012588 | Novgorod | Modern | Hirata et al. 2013 | 16962 |
| 3a | AP012589 | Novgorod | Modern | Hirata et al. 2013 | 16962 |
| 3a | AP012590.1 | Balkan Mountains, Bulgaria | Modern | Hirata et al. 2013 | 16962 |
| 3a | AP012579 | Sakhalin, Japan | Modern | Hirata et al. 2013 | 16945 |
| 3a | EU574910 | Alaska | Modern | Jackson et al. 2008 | 589 |
| 3a | EU574911 | Alaska | Modern | Jackson et al. 2008 | 589 |
| 3a | HQ685901 | North-Estonia | Modern | Keis et al. 2012 | 16793 |
| 3a | HQ685910 | North-Estonia | Modern | Keis et al. 2012 | 16793 |
| 3a | HQ685911 | North-Estonia | Modern | Keis et al. 2012 | 16793 |
| 3a | HQ685912 | South-Estonia , North-Estonia | Modern | Keis et al. 2012 | 16793 |
| 3a | HQ685913 | South-Estonia | Modern | Keis et al. 2012 | 16793 |
| 3a | HQ685914 | North-Estonia | Modern | Keis et al. 2012 | 16793 |
| 3a | HQ685915 | North-Estonia | Modern | Keis et al. 2012 | 16793 |
| 3a | HQ685916 | Western-Russia | Modern | Keis et al. 2012 | 16793 |
| 3a | HQ685917 | Western-Russia | Modern | Keis et al. 2012 | 16793 |
| 3a | HQ685918 | Western-Russia | Modern | Keis et al. 2012 | 16793 |
| 3a | HQ685919 | Western-Russia | Modern | Keis et al. 2012 | 16793 |
| 3a | HQ685902 | South-Estonia , North-Estonia | Modern | Keis et al. 2012 | 16793 |
| 3a | HQ685920 | Western-Russia | Modern | Keis et al. 2012 | 16793 |
| 3a | HQ685921 | Western-Russia | Modern | Keis et al. 2012 | 16793 |
| 3a | HQ685922 | Western-Russia | Modern | Keis et al. 2012 | 16793 |
| 3a | HQ685923 | Western-Russia | Modern | Keis et al. 2012 | 16793 |
| 3a | HQ685924 | Western-Russia | Modern | Keis et al. 2012 | 16793 |
| 3a | HQ685925 | Western-Russia | Modern | Keis et al. 2012 | 16793 |
| 3a | HQ685926 | Western-Russia | Modern | Keis et al. 2012 | 16793 |
| 3a | HQ685927 | Northern-Russia | Modern | Keis et al. 2012 | 16793 |
| 3a | HQ685928 | Northern-Russia | Modern | Keis et al. 2012 | 16793 |
| 3a | HQ685929 | Northern-Russia | Modern | Keis et al. 2012 | 16793 |
| 3a | HQ685903 | North-Estonia | Modern | Keis et al. 2012 | 16793 |
| 3a | HQ685930 | Northern-Russia | Modern | Keis et al. 2012 | 16793 |
| 3a | HQ685931 | Northern-Russia | Modern | Keis et al. 2012 | 16793 |
| 3a | HQ685932 | Northern-Russia | Modern | Keis et al. 2012 | 16793 |
| 3a | HQ685933 | Northern-Russia | Modern | Keis et al. 2012 | 16793 |
| 3a | HQ685934 | Northern-Russia | Modern | Keis et al. 2012 | 16793 |
| 3a | HQ685935 | Northern-Russia | Modern | Keis et al. 2012 | 16793 |
| 3a | HQ685936 | Northern-Russia | Modern | Keis et al. 2012 | 16793 |
| 3a | HQ685937 | Eastern-Russia | Modern | Keis et al. 2012 | 16793 |
| 3a | HQ685938 | Eastern-Russia | Modern | Keis et al. 2012 | 16793 |
| 3a | HQ685939 | Eastern-Russia | Modern | Keis et al. 2012 | 16793 |
| 3a | HQ685904 | North-Estonia | Modern | Keis et al. 2012 | 16793 |
| 3a | HQ685940 | Eastern-Russia | Modern | Keis et al. 2012 | 16793 |
| 3a | HQ685941 | Eastern-Russia | Modern | Keis et al. 2012 | 16793 |
| 3a | HQ685942 | Eastern-Russia | Modern | Keis et al. 2012 | 16793 |
| 3a | HQ685943 | Eastern-Russia | Modern | Keis et al. 2012 | 16793 |
| 3a | HQ685944 | Eastern-Russia | Modern | Keis et al. 2012 | 16793 |
| 3a | HQ685945 | Eastern-Russia | Modern | Keis et al. 2012 | 16793 |
| 3a | HQ685946 | Eastern-Russia | Modern | Keis et al. 2012 | 16793 |
| 3a | HQ685947 | Western-Russia | Modern | Keis et al. 2012 | 16793 |
| 3a | HQ685948 | Western-Russia | Modern | Keis et al. 2012 | 16793 |
| 3a | HQ685949 | Western-Russia | Modern | Keis et al. 2012 | 16793 |
| 3a | HQ685905 | North-Estonia | Modern | Keis et al. 2012 | 16793 |
| 3a | HQ685950 | Central-Russia | Modern | Keis et al. 2012 | 16793 |
| 3a | HQ685951 | Central-Russia | Modern | Keis et al. 2012 | 16793 |
| 3a | HQ685952 | Central-Russia | Modern | Keis et al. 2012 | 16793 |
| 3a | HQ685953 | Central-Russia | Modern | Keis et al. 2012 | 16793 |
| 3a | HQ685954 | Western-Russia | Modern | Keis et al. 2012 | 16793 |
| 3a | HQ685955 | Northern-Finland | Modern | Keis et al. 2012 | 16793 |
| 3a | HQ685956 | Southern-Finland | Modern | Keis et al. 2012 | 16793 |
| 3a | HQ685957 | Southern-Finland, Northern-Finland | Modern | Keis et al. 2012 | 16793 |
| 3a | HQ685958 | Southern-Finland | Modern | Keis et al. 2012 | 16793 |
| 3a | HQ685959 | Southern-Finland | Modern | Keis et al. 2012 | 16793 |
| 3a | HQ685906 | South-Estonia , North-Estonia | Modern | Keis et al. 2012 | 16793 |
| 3a | HQ685960 | Northern-Finland | Modern | Keis et al. 2012 | 16793 |
| 3a | HQ685961 | Southern-Finland | Modern | Keis et al. 2012 | 16793 |
| 3a | HQ685962 | Northern-Finland | Modern | Keis et al. 2012 | 16793 |
| 3a | HQ685963 | Northern-Finland | Modern | Keis et al. 2012 | 16793 |
| 3a | HQ685964 | Southern-Finland | Modern | Keis et al. 2012 | 16793 |
| 3a | HQ685907 | North-Estonia | Modern | Keis et al. 2012 | 16793 |
| 3a | HQ685908 | North-Estonia | Modern | Keis et al. 2012 | 16793 |
| 3a | HQ685909 | South-Estonia | Modern | Keis et al. 2012 | 16793 |
| 3a | EU526803 | KA1, Kamchatka | Modern | Korsten et al. 2009 | 663 |
| 3a | EU526812 | KA10, Kamchatka | Modern | Korsten et al. 2009 | 662 |
| 3a | EU526813 | KA11, Kamchatka | Modern | Korsten et al. 2009 | 663 |
| 3a | EU526814 | KA12, Kamchatka | Modern | Korsten et al. 2009 | 663 |
| 3a | EU526804 | KA2, Kamchatka | Modern | Korsten et al. 2009 | 664 |
| 3a | EU526805 | KA3, Kamchatka | Modern | Korsten et al. 2009 | 664 |
| 3a | EU526806 | KA4, Kamchatka | Modern | Korsten et al. 2009 | 664 |
| 3a | EU526807 | KA5, Kamchatka | Modern | Korsten et al. 2009 | 663 |
| 3a | EU526808 | KA6, Kamchatka | Modern | Korsten et al. 2009 | 663 |
| 3a | EU526809 | KA7, Kamchatka | Modern | Korsten et al. 2009 | 663 |
| 3a | EU526810 | KA8, Kamchatka | Modern | Korsten et al. 2009 | 663 |
| 3a | EU526811 | KA9, Kamchatka | Modern | Korsten et al. 2009 | 663 |
| 3a | EU526765 | EA1, Estonia, Finland, European Russia, Far East Russia | Modern | Korsten et al. 2009 | 663 |
| 3a | EU526774 | EA10, European Russia | Modern | Korsten et al. 2009 | 666 |
| 3a | EU526775 | EA11, Far East Russia | Modern | Korsten et al. 2009 | 663 |
| 3a | EU526776 | EA12, European Russia | Modern | Korsten et al. 2009 | 664 |
| 3a | EU526777 | EA13, Finland, European Russia | Modern | Korsten et al. 2009 | 663 |
| 3a | EU526778 | EA14, Finland | Modern | Korsten et al. 2009 | 666 |
| 3a | EU526779 | EA15, Finland | Modern | Korsten et al. 2009 | 663 |
| 3a | EU526780 | EA16, Finland | Modern | Korsten et al. 2009 | 664 |
| 3a | EU526781 | EA17, European Russia | Modern | Korsten et al. 2009 | 663 |
| 3a | EU526782 | EA18, European Russia | Modern | Korsten et al. 2009 | 665 |
| 3a | EU526783 | EA19, European Russia | Modern | Korsten et al. 2009 | 663 |
| 3a | EU526766 | EA2, European Russia | Modern | Korsten et al. 2009 | 663 |
| 3a | EU526784 | EA20, Estonia | Modern | Korsten et al. 2009 | 663 |
| 3a | EU526785 | EA21, Estonia, European Russia | Modern | Korsten et al. 2009 | 663 |
| 3a | EU526786 | EA22, European Russia | Modern | Korsten et al. 2009 | 667 |
| 3a | EU526787 | EA23, European Russia | Modern | Korsten et al. 2009 | 664 |
| 3a | EU526788 | EA24, European Russia | Modern | Korsten et al. 2009 | 663 |
| 3a | EU526789 | EA25, European Russia | Modern | Korsten et al. 2009 | 664 |
| 3a | EU526790 | EA26, Siberia | Modern | Korsten et al. 2009 | 663 |
| 3a | EU526791 | EA27, European Russia | Modern | Korsten et al. 2009 | 663 |
| 3a | EU526792 | EA28, Finland | Modern | Korsten et al. 2009 | 662 |
| 3a | EU526767 | EA3, Finland | Modern | Korsten et al. 2009 | 667 |
| 3a | EU526768 | EA4, European Russia | Modern | Korsten et al. 2009 | 664 |
| 3a | EU526769 | EA5, European Russia | Modern | Korsten et al. 2009 | 664 |
| 3a | EU526770 | EA6, Finland, European Russia | Modern | Korsten et al. 2009 | 663 |
| 3a | EU526771 | EA7, European Russia | Modern | Korsten et al. 2009 | 663 |
| 3a | EU526772 | EA8, European Russia | Modern | Korsten et al. 2009 | 663 |
| 3a | EU526773 | EA9, Finland | Modern | Korsten et al. 2009 | 663 |
| 3a | EU526793 | EB1, Finland, Europeand Russia, Siberia | Modern | Korsten et al. 2009 | 664 |
| 3a | EU526794 | EB2, Siberia | Modern | Korsten et al. 2009 | 664 |
| 3a | EU526795 | EB3, Fast East Russia | Modern | Korsten et al. 2009 | 666 |
| 3a | EU526796 | EB4, Fast East Russia | Modern | Korsten et al. 2009 | 667 |
| 3a | EU526797 | EB5, Kamchatka | Modern | Korsten et al. 2009 | 664 |
| 3a | EU526798 | EB6, Kamchatka | Modern | Korsten et al. 2009 | 666 |
| 3a | EU526799 | EB7, Finland | Modern | Korsten et al. 2009 | 663 |
| 3a | EU526800 | EB8, Siberia | Modern | Korsten et al. 2009 | 665 |
| 3a | EU526801 | EC1, Estonia | Modern | Korsten et al. 2009 | 663 |
| 3a | EU526802 | EC2, Estonia | Modern | Korsten et al. 2009 | 663 |
| 3 | AB010725 | Hokaido (Japan) | Modern | Masuda et al. 1998 | 268 |
| 3 | AB010726 | Hokaido (Japan) | Modern | Masuda et al. 1998 | 267 |
| 3 | AB013040 | HB-01 (Japan) | Modern | Matsuhashi et al. 1999 | 697 |
| 3 | AB013045 | HB-02 (Japan) | Modern | Matsuhashi et al. 1999 | 701 |
| 3 | AB013046 | HB-03 (Japan) | Modern | Matsuhashi et al. 1999 | 697 |
| 3 | AB013049 | HB-04 (Japan) | Modern | Matsuhashi et al. 1999 | 700 |
| 3 | AB013051 | HB-05 (Japan) | Modern | Matsuhashi et al. 1999 | 698 |
| 3 | AB013052 | HB-06 (Japan) | Modern | Matsuhashi et al. 1999 | 698 |
| 3 | AB013053 | HB-07 (Japan) | Modern | Matsuhashi et al. 1999 | 697 |
| 3 | AB013054 | HB-08 (Japan) | Modern | Matsuhashi et al. 1999 | 697 |
| 3 | AB013055 | HB-09 (Japan) | Modern | Matsuhashi et al. 1999 | 696 |
| 3 | AB013056 | HB-10 (Japan) | Modern | Matsuhashi et al. 1999 | 698 |
| 3 | AB013057 | HB-11 (Japan) | Modern | Matsuhashi et al. 1999 | 697 |
| 3 | AB013058 | HB-12 (Japan) | Modern | Matsuhashi et al. 1999 | 697 |
| 3 | AB013060 | HB-13 (Japan) | Modern | Matsuhashi et al. 1999 | 698 |
| 3 | AB013061 | HB-14 (Japan) | Modern | Matsuhashi et al. 1999 | 700 |
| 3 | AB013062 | HB-15 (Japan) | Modern | Matsuhashi et al. 1999 | 698 |
| 3 | AB013064 | HB-16 (Japan) | Modern | Matsuhashi et al. 1999 | 701 |
| 3 | AB013070 | HB-17 (Japan) | Modern | Matsuhashi et al. 1999 | 698 |
| 3 | AB041258 | Kodiak (Zoo Japan) | Modern | Matsuhashi et al. 1999 | 268 |
| 3a | GU057344 | Georgia | Modern | Murtskhvaladze et al. 2010 | 351 |
| 3a | GU057345 | Georgia | Modern | Murtskhvaladze et al. 2010 | 351 |
| 3a | GU057346 | Georgia | Modern | Murtskhvaladze et al. 2010 | 351 |
| 3a | GU057347 | Georgia | Modern | Murtskhvaladze et al. 2010 | 351 |
| 3a | GU057343 | Georgia | Modern | Murtskhvaladze et al. 2010 | 351 |
| 3a | GU057356 | Georgia | Modern | Murtskhvaladze et al. 2010 | 351 |
| 3a | GU057358 | Georgia | Modern | Murtskhvaladze et al. 2010 | 351 |
| 3a | GU057359 | Georgia | Modern | Murtskhvaladze et al. 2010 | 351 |
| 3a | GU057361 | Georgia | Modern | Murtskhvaladze et al. 2010 | 351 |
| 3a | GU057362 | Georgia | Modern | Murtskhvaladze et al. 2010 | 351 |
| 3a | GU057364 | Georgia | Modern | Murtskhvaladze et al. 2010 | 351 |
| 3a | GU057373 | Georgia | Modern | Murtskhvaladze et al. 2010 | 351 |
| 3a | GU057367 | Georgia | Modern | Murtskhvaladze et al. 2010 | 351 |
| 3a | GU057368 | Georgia | Modern | Murtskhvaladze et al. 2010 | 351 |
| 3a | GU057369 | Georgia | Modern | Murtskhvaladze et al. 2010 | 351 |
| 3a | GU057371 | Georgia | Modern | Murtskhvaladze et al. 2010 | 351 |
| 3a | GU057372 | Georgia | Modern | Murtskhvaladze et al. 2010 | 351 |
| 3a | GU057375 | Georgia | Modern | Murtskhvaladze et al. 2010 | 351 |
| 3a | GU057376 | Georgia | Modern | Murtskhvaladze et al. 2010 | 351 |
| 3a | GU057377 | Georgia | Modern | Murtskhvaladze et al. 2010 | 351 |
| 3a | GU057351 | Georgia | Modern | Murtskhvaladze et al. 2010 | 351 |
| 3a | GU057349 | Georgia | Modern | Murtskhvaladze et al. 2010 | 351 |
| 3a | EF488498 | Mühlberg (Thuringia, Germany) | 1690 ± 54 | Valdiosera et al. 2007 | 193 |
| 3a | KM886400 | Jämtland, Norrbotten, Västerbotten (Sweden) | 1832 - 1953 | Xenikoudakis et al. 2015 | 265 |
| 3a | KM886405 | Nordland fylke (Norway); Jämtland, Norrbotten, Västerbotten (Sweden) | 1915 - 1940 | Xenikoudakis et al. 2015 | 266 |
| 3a | KM886419 | Västerbotten (Sweden) | 1915 | Xenikoudakis et al. 2015 | 268 |

**References:**

1. Baca, M. et al. Ancient DNA and dating of cave bear remains from Niedźwiedzia Cave suggest early appearance of Ursus ingressus in Sudetes. *Quat. Int.* **339**, 217-223 (2014).
2. Barnes, I., Matheus, P., Shapiro, B., Jensen, D. & Cooper, A. Dynamics of Pleistocene Population Extinctions in Beringian Brown Bears. *Science* **295**, 2267–2270 (2002).
3. Hirata, D. et al. Molecular phylogeography of the brown bear (*Ursus arctos*) in Northeastern Asia based on analyses of complete mitochondrial DNA sequences. *Mol. Biol. Evol.* **30**, 1644–1652 (2013).
4. Keis, M. et al. Complete mitochondrial genomes and a novel spatial genetic method reveal cryptic phylogeographical structure and migration patterns among brown bears in north‐western Eurasia. *J. Biogeogr.* **40**, 915-927 (2013).
5. Korsten, M. et al. Sudden expansion of a single brown bear maternal lineage across northern continental Eurasia after the last ice age: a general demographic model for mammals? *Mol. Ecol.* **18**, 1963–1979 (2009).
6. Masuda, R., Murata, K., Aiurzaniin, A. & Yoshida, M. C. Phylogenetic status of brown bears *Ursus arctos* of Asia: a preliminary result inferred from mitochondrial DNA control region sequences. *Hereditas* **128**, 277-280 (1998).
7. Matsuhashi T., Masuda R., Mano T. & Yoshida M.C. Microevolution of the mitochondrial DNA control region in the Japanese brown bear (*Ursus arctos*) population. *Mol. Biol. Evol.*, **16**, 676–84 (1999).
8. Murtskhvaladze, M., Gavashelishvili, A. & Tarkhnishvili, D. Geographic and genetic boundaries of brown bear (*Ursus arctos*) population in the Caucasus. *Mol. Ecol.* **19**, 1829-1841 (2010).
9. Xenikoudakis, G. et al. Consequences of a demographic bottleneck on genetic structure and variation in the Scandinavian brown bear. *Mol. Ecol.* **24**, 3441-3454 (2015).
10. Valdiosera, C.E. et al. Surprising migration and population size dynamics in ancient Iberian brown bears (Ursus arctos).  *Proc. Natl. Acad. Sci. U.S.A.* **105**, 5123–5128 (2008).

Supplementary Table 5. Database of stable isotopes (*δ*^15^N and *δ*^13^C) from Eastern and Western Beringia.

| **Sample** | **Species** | **Area** | **Datation** | **δ^15^N** | **δ^13^C** | **Reference** |
| --- | --- | --- | --- | --- | --- | --- |
| IK01-074 | Bison | E Beringia | >38000 | 5,8 | -20,0 | 1 |
| IK99-501 | Bison | E Beringia | >39000 | 4,0 | -20,4 | 1 |
| IK99-145 | Bison | E Beringia | >39400 | 4,0 | -19,1 | 1 |
| IK01-315 | Bison | E Beringia | >40000 | 7,6 | -19,5 | 1 |
| IK01-098 | Bison | E Beringia | >40700 | 5,9 | -19,7 | 1 |
| IK01-143 | Bison | E Beringia | >40900 | 6,6 | -20,0 | 1 |
| IK01-095 | Bison | E Beringia | >41000 | 4,9 | -19,2 | 1 |
| IK01-260 | Bison | E Beringia | >41000 | 6,5 | -19,8 | 1 |
| IK99-141 | Bison | E Beringia | >41100 | 2,8 | -19,2 | 1 |
| IK01-065 | Bison | E Beringia | >41100 | 4,3 | -20,4 | 1 |
| IK99-530 | Bison | E Beringia | >41100 | 6,9 | -19,8 | 1 |
| IK01-088 | Bison | E Beringia | >41100 | 6,5 | -20,2 | 1 |
| IK99-567 | Bison | E Beringia | >41500 | 6,2 | -20,3 | 1 |
| IK12-001 | Bison | E Beringia | >43500 | 4,2 | -20,0 | 1 |
| IK98-0218 | Bison | E Beringia | >46600 | 2,7 | -20,4 | 1 |
| IK98-1041 | Bison | E Beringia | >48500 | 4,0 | -19,5 | 1 |
| IK98-1299 | Bison | E Beringia | >49500 | 3,3 | -20,1 | 1 |
| IK98-1167 | Bison | E Beringia | >49900 | 4,9 | -20,2 | 1 |
| IK98-1015 | Bison | E Beringia | >49900 | 2,0 | -20,4 | 1 |
| IK98-1090 | Bison | E Beringia | 21040 ± 120 | 6,5 | -20,0 | 1 |
| IK98-0401 | Bison | E Beringia | 21530 ± 130 | 4,4 | -20,4 | 1 |
| IK98-1254 | Bison | E Beringia | 23680 ± 170 | 5,5 | -19,9 | 1 |
| IK98-0302 | Bison | E Beringia | 24500 ± 180 | 4,3 | -20,2 | 1 |
| IK98-1184 | Bison | E Beringia | 25980 ± 230 | 4,2 | -19,7 | 1 |
| IK98-1043 | Bison | E Beringia | 26550 ± 230 | 5,7 | -19,5 | 1 |
| IK98-0095 | Bison | E Beringia | 27400 ± 260 | 4,6 | -20,6 | 1 |
| IK98-0374 | Bison | E Beringia | 27590 ± 280 | 2,7 | -20,5 | 1 |
| IK98-1115 | Bison | E Beringia | 28120 ± 290 | 4,8 | -19,3 | 1 |
| IK98-0616 | Bison | E Beringia | 29040 ± 340 | 5,2 | -20,2 | 1 |
| IK98-1164 | Bison | E Beringia | 29570 ± 340 | 5,0 | -20,0 | 1 |
| IK98-0256 | Bison | E Beringia | 31410 ± 420 | 4,2 | -20,4 | 1 |
| IK98-0096 | Bison | E Beringia | 31630 ± 440 | 4,9 | -19,9 | 1 |
| IK98-1035 | Bison | E Beringia | 32270 ± 470 | 5,3 | -19,6 | 1 |
| IK01-215 | Bison | E Beringia | 32300 ± 1500 | 4,4 | -20,2 | 1 |
| IK01-433 | Bison | E Beringia | 33000 ± 1500 | 3,5 | -19,9 | 1 |
| IK98-0012 | Bison | E Beringia | 33280 ± 530 | 3,5 | -19,4 | 1 |
| IK98-1323 | Bison | E Beringia | 33320 ± 540 | 4,4 | -20,2 | 1 |
| IK01-460 | Bison | E Beringia | 33520 ± 940 | 5,1 | -19,9 | 1 |
| IK98-1121 | Bison | E Beringia | 33580 ± 550 | 3,8 | -19,6 | 1 |
| IK01-234 | Bison | E Beringia | 34100 ± 1000 | 5,9 | -20,0 | 1 |
| IK98-0659 | Bison | E Beringia | 35580 ± 720 | 4,6 | -20,0 | 1 |
| IK98-0916 | Bison | E Beringia | 35710 ± 730 | 4,2 | -20,3 | 1 |
| IK98-1222 | Bison | E Beringia | 36320 ± 780 | 5,7 | -20,8 | 1 |
| IK01-373 | Bison | E Beringia | 36500 ± 2300 | 6,4 | -20,0 | 1 |
| IK98-0863 | Bison | E Beringia | 36520 ± 800 | 4,5 | -20,6 | 1 |
| IK98-1120 | Bison | E Beringia | 37460 ± 890 | 6,7 | -20,7 | 1 |
| IK98-0377 | Bison | E Beringia | 38700 ± 1000 | 4,5 | -19,9 | 1 |
| IK98-0889 | Bison | E Beringia | 38800 ± 1100 | 3,7 | -20,3 | 1 |
| IK98-0890 | Bison | E Beringia | 38800 ± 1100 | 5,3 | -20,4 | 1 |
| IK98-0915 | Bison | E Beringia | 39800 ± 1200 | 4,5 | -20,3 | 1 |
| IK98-0174 | Bison | E Beringia | 39850 ± 1200 | 4,5 | -20,4 | 1 |
| IK98-1122 | Bison | E Beringia | 40700 ± 1300 | 4,3 | -20,0 | 1 |
| IK98-1045 | Bison | E Beringia | 43000 ± 1800 | 4,2 | -20,2 | 1 |
| IK98-1042 | Bison | E Beringia | 44800 ± 2200 | 3,5 | -19,6 | 1 |
| IK98-1125 | Bison | E Beringia | 45300 ± 2400 | 4,5 | -20,0 | 1 |
| IK98-0032 | Bison | E Beringia | 46100 ± 2200 | 3,3 | -20,6 | 1 |
| IK98-0305 | Bison | E Beringia | 46100 ± 2600 | 5,7 | -20,1 | 1 |
| IK98-0671 | Bison | E Beringia | 47000 ± 2900 | 6,2 | -20,5 | 1 |
| IK98-0527 | Bison | E Beringia | 50000 ± 4200 | 2,7 | -20,7 | 1 |
| 17/1 | Bison | W Beringia | 40000 | 9,8 | -21,6 | 2 |
| B, priscus 25 | Bison | W Beringia | 38200 ± 700 | 7,4 | -20,7 | 3 |
| B, priscus 26 | Bison | W Beringia | 41500 ± 1100 | 5,2 | -20,1 | 3 |
| F-3246-5 | Bison | W Beringia |  | 8 | -21,9 | 4 |
| F-3246-116 | Bison | W Beringia |  | 7,7 | -21,3 | 4 |
| F-3246-117 | Bison | W Beringia |  | 7,7 | -21,3 | 4 |
| F-3080 | Bison | W Beringia |  | 6,4 | -21 | 4 |
| F-3079 | Bison | W Beringia |  | 8,3 | -20,8 | 4 |
| F-3077-1 | Bison | W Beringia |  | 7,2 | -20,7 | 4 |
| F-3009 | Bison | W Beringia |  | 7,1 | -21,4 | 4 |
| F-3010 | Bison | W Beringia |  | 7,8 | -20,9 | 4 |
| F-3008 | Bison | W Beringia |  | 5,6 | -21,3 | 4 |
| F-3011 | Bison | W Beringia |  | 7,5 | -21,5 | 4 |
| F-3076 | Bison | W Beringia |  | 8 | -20,9 | 4 |
| F-2884-3 | Bison | W Beringia |  | 7,6 | -21,3 | 4 |
| AMNH F:AM 95640 | Brown bear | E Beringia | 53900 | 6,4 | -19,6 | 5 |
| AMNH F:AM 95639 | Brown bear | E Beringia | 56900 | 6,8 | -19,0 | 5 |
| AMNH F:AM 95681 | Brown bear | E Beringia | 59000 | 6,0 | -19,4 | 5 |
| AMNH F:AM 95666 | Brown bear | E Beringia | 47100±3100 | 6,3 | -19,5 | 5 |
| AMNH F:AM 95609 | Brown bear | E Beringia | 50800±1900 | 7,7 | -19,2 | 5 |
| FAM 30771 | Brown bear | E Beringia | 20080 ± 160 | 7,4 | -19,8 | 5 |
| FAM 95671 | Brown bear | E Beringia | 20820 ± 120 | 6,1 | -18,9 | 5 |
| CMN 38279 | Brown bear | E Beringia | 36500 ± 1150 | 6,7 | -20,0 | 5; 6 |
| CMN 35965 | Brown bear | E Beringia | 41000 ± 1050 | 4,4 | -19,8 | 5; 6 |
| PM collected | Brown bear | E Beringia | 48164 ± 3224 | 9,1 | -19,1 | 5; 6 |
| AMNH F:AM 95601 | Brown bear | E Beringia | 36137±783 | 3,3 | -20,0 | 5; 6 |
| F-2374 | Brown bear | W Beringia |  | 12,3 | -19,6 | 4 |
| F-2723 | Brown bear | W Beringia |  | 9 | -20,2 | 4 |
| F-931 | Brown bear | W Beringia |  | 12,8 | -19 | 4 |
| F-2296 | Brown bear | W Beringia |  | 11,8 | -20,1 | 4 |
| DGI-1 | Brown bear | W Beringia |  | 11,8 | -20,2 | This study |
| CGG_1_0200005 / DGI-2 | Brown bear | W Beringia |  | 12,0 | -19,9 | This study |
| CGG_1_0200006 / DGI-3 | Brown bear | W Beringia |  | 10,4 | -19,8 | This study |
| DGI-4 | Brown bear | W Beringia |  | 9,3 | -19,8 | This study |
| CGG_1_0200007 /DGI-5 | Brown bear | W Beringia |  | 9,65 | -21,6 | This study |
| AMNH F:AM 60028 | Horse | E Beringia | 48400 | 4,4 | -20,6 | 7 |
| AMNH F:AM 60019 | Horse | E Beringia | 48500 | 1,7 | -21,5 | 7 |
| AMNH F:AM 60003 | Horse | E Beringia | 24260±200 | 1,4 | -21,3 | 7 |
| AMNH F:AM 60033 | Horse | E Beringia | 39910±1330 | 2,6 | -20,9 | 7 |
| AMNH F:AM 60017 | Horse | E Beringia | 41000±1500 | 3,6 | -21,6 | 7 |
| AMNH F:AM 60221 | Horse | E Beringia | 43700±2000 | 0,7 | -21,7 | 7 |
| AMNH F:AM 60028 | Horse | E Beringia | >48400 | 4,4 | -20,6 | 7 |
| AMNH F:AM 142422 | Horse | E Beringia | >48500 | 3,0 | -21,5 | 7 |
| AMNH F:AM 142432 | Horse | E Beringia | >48500 | 2,5 | -21,5 | 7 |
| AMNH F:AM 60019 | Horse | E Beringia | >48500 | 1,7 | -21,5 | 7 |
| AMNH F:AM 142434 | Horse | E Beringia | 22610 ± 150 | 3,8 | -21,0 | 7 |
| AMNH F:AM 60003 | Horse | E Beringia | 24260 ± 200 | 1,4 | -21,3 | 7 |
| AMNH F:AM 142431 | Horse | E Beringia | 25710 ± 230 | 3,9 | -21,1 | 7 |
| AMNH F:AM 142433 | Horse | E Beringia | 25960 ± 240 | 2,9 | -20,7 | 7 |
| AMNH F:AM 60033 | Horse | E Beringia | 39910 ± 1330 | 2,6 | -20,9 | 7 |
| AMNH F:AM 60017 | Horse | E Beringia | 41000 ± 1500 | 3,6 | -21,6 | 7 |
| AMNH F:AM 60221 | Horse | E Beringia | 43700 ± 2000 | 0,7 | -21,7 | 7 |
| IK02-072 | Horse | E Beringia | >43500 | 7,7 | -21,3 | 1 |
| IK01-320 | Horse | E Beringia | 23230 ± 90 | 5,6 | -20,9 | 1 |
| IK01-320 | Horse | E Beringia | 23790 ± 110 | 5,6 | -20,9 | 1 |
| IK08-080 | Horse | E Beringia | 24070 ± 100 | 4,8 | -21,1 | 1 |
| IK99-129 | Horse | E Beringia | 24690 ± 110 | 4,9 | -20,3 | 1 |
| T02-001 | Horse | E Beringia | 24900 ± 100 | 3,7 | -20,8 | 1 |
| IK99-367 | Horse | E Beringia | 25680 ± 140 | 5,3 | -20,6 | 1 |
| IK01-218 | Horse | E Beringia | 26020 ± 120 | 6,0 | -20,9 | 1 |
| IK12-063 | Horse | E Beringia | 26130 ± 120 | 7,7 | -20,5 | 1 |
| IK01-218 | Horse | E Beringia | 26190 ± 120 | 6,0 | -20,9 | 1 |
| IK99-367 | Horse | E Beringia | 26210 ± 130 | 5,3 | -20,6 | 1 |
| KIK08-01 | Horse | E Beringia | 26460 ± 130 | 4,6 | -21,6 | 1 |
| IK11-001 | Horse | E Beringia | 26770 ± 140 | 4,5 | -20,8 | 1 |
| TIT11-070 | Horse | E Beringia | 26890 ± 150 | 7,4 | -20,6 | 1 |
| IK01-080 | Horse | E Beringia | 27060 ± 140 | 5,4 | -21,1 | 1 |
| IK12-015 | Horse | E Beringia | 27810 ± 210 | 6,4 | -20,7 | 1 |
| IK01-459 | Horse | E Beringia | 27930 ± 150 | 7,6 | -20,9 | 1 |
| IK10-074 | Horse | E Beringia | 28260 ± 210 | 5,0 | -20,6 | 1 |
| IK98-1176 | Horse | E Beringia | 28330 ± 150 | 5,1 | -20,9 | 1 |
| T04-004 | Horse | E Beringia | 28500 ± 160 | 6,1 | -21,3 | 1 |
| IK99-244 | Horse | E Beringia | 28500 ± 200 | 8,2 | -20,6 | 1 |
| IK01-080 | Horse | E Beringia | 28540 ± 170 | 5,4 | -21,1 | 1 |
| IK01-368 | Horse | E Beringia | 28600 ± 200 | 7,6 | -21,2 | 1 |
| IK06-17 | Horse | E Beringia | 28690 ± 160 | 6,5 | -20,9 | 1 |
| IK99-254 | Horse | E Beringia | 29560 ± 150 | 9,8 | -21,2 | 1 |
| IK99-383 | Horse | E Beringia | 29700 ± 200 | 6,4 | -21,3 | 1 |
| TIT11-071 | Horse | E Beringia | 29830 ± 190 | 6,8 | -20,9 | 1 |
| IK07-06 | Horse | E Beringia | 30260 ± 190 | 5,8 | -21,1 | 1 |
| IK99-383 | Horse | E Beringia | 30560 ± 160 | 6,4 | -21,3 | 1 |
| TIT11-072 | Horse | E Beringia | 30560 ± 200 | 8,1 | -21,7 | 1 |
| IK98-0539 | Horse | E Beringia | 30610 ± 200 | 7,9 | -21,2 | 1 |
| IK01-183 | Horse | E Beringia | 30900 ± 300 | 5,3 | -21,7 | 1 |
| IK08-082 | Horse | E Beringia | 31230 ± 270 | 7,4 | -21,0 | 1 |
| TIT10-38 | Horse | E Beringia | 31680 ± 190 | 5,1 | -21,3 | 1 |
| IK98-1142 | Horse | E Beringia | 32250 ± 220 | 5,1 | -21,1 | 1 |
| IK01-282 | Horse | E Beringia | 32600 ± 300 | 4,5 | -20,9 | 1 |
| IK09-51 | Horse | E Beringia | 32700 ± 300 | 5,5 | -21,6 | 1 |
| TIT10-36 | Horse | E Beringia | 33150 ± 200 | 6,2 | -21,4 | 1 |
| IK98-0288 | Horse | E Beringia | 33200 ± 240 | 6,8 | -21,5 | 1 |
| IK98-0112 | Horse | E Beringia | 33420 ± 340 | 7,9 | -21,1 | 1 |
| IK98-0288 | Horse | E Beringia | 33800 ± 400 | 6,8 | -21,5 | 1 |
| IK99-790 | Horse | E Beringia | 33820 ± 290 | 5,9 | -20,7 | 1 |
| IK01-369 | Horse | E Beringia | 33870 ± 350 | 5,9 | -21,2 | 1 |
| IK01-369 | Horse | E Beringia | 33900 ± 400 | 5,9 | -21,2 | 1 |
| IK01-150 | Horse | E Beringia | 34200 ± 400 | 7,2 | -21,2 | 1 |
| TIT10-37 | Horse | E Beringia | 34210 ± 370 | 9,1 | -20,9 | 1 |
| IK99-404 | Horse | E Beringia | 34320 ± 270 | 8,3 | -22,1 | 1 |
| IK99-404 | Horse | E Beringia | 34690 ± 340 | 8,3 | -22,1 | 1 |
| IK98-0009 | Horse | E Beringia | 35380 ± 360 | 5,9 | -21,0 | 1 |
| IK98-0394 | Horse | E Beringia | 35500 ± 400 | 6,0 | -21,4 | 1 |
| IK01-121 | Horse | E Beringia | 36500 ± 500 | 6,8 | -21,2 | 1 |
| IK99-806 | Horse | E Beringia | 37400 ± 540 | 5,8 | -20,8 | 1 |
| IK08-079 | Horse | E Beringia | 38090 ± 590 | 5,7 | -21,2 | 1 |
| IK08-078 | Horse | E Beringia | 40880 ± 400 | 7,0 | -20,6 | 1 |
| IK99-111 | Horse | E Beringia | 40960 ± 370 | 3,1 | -20,8 | 1 |
| IK12-011 | Horse | E Beringia | 41000 ± 800 | 4,2 | -20,9 | 1 |
| IK12-010 | Horse | E Beringia | 41410 ± 570 | 4,3 | -20,4 | 1 |
| IK02-072 | Horse | E Beringia | 41840 ± 410 | 7,7 | -21,3 | 1 |
| KIK12-02 | Horse | E Beringia | 42500 ± 1000 | 5,7 | -21,0 | 1 |
| IK02-026 | Horse | E Beringia | 43700 ± 1000 | 1,7 | -20,7 | 1 |
| 96300 | Horse | W Beringia | 25000 | 4,3 | -21,1 | 8 |
| E, Caballus 24 | Horse | W Beringia | 35900 ± 600 | 4,4 | -20,8 | 3 |
| F-2993 | Horse | W Beringia |  | 6,7 | -21,1 | 4 |
| F-2988 | Horse | W Beringia |  | 6,6 | -20,9 | 4 |
| F-2979 | Horse | W Beringia |  | 5,4 | -21,3 | 4 |
| F-2987 | Horse | W Beringia |  | 6,2 | -21,2 | 4 |
| F-2982 | Horse | W Beringia |  | 5,8 | -21 | 4 |
| F-2972 | Horse | W Beringia |  | 5,9 | -21 | 4 |
| F-2989 | Horse | W Beringia |  | 6,1 | -21,3 | 4 |
| F-2990 | Horse | W Beringia |  | 3,7 | -21 | 4 |
| F-2977 | Horse | W Beringia |  | 5,5 | -20,7 | 4 |
| F-3002 | Horse | W Beringia |  | 7,7 | -21,1 | 4 |
| F-2969 | Horse | W Beringia |  | 7,7 | -20,5 | 4 |
| F-2971 | Horse | W Beringia |  | 6,6 | -21,1 | 4 |
| F-2976 | Horse | W Beringia |  | 9 | -22 | 4 |
| F-2968 | Horse | W Beringia |  | 5,6 | -21,1 | 4 |
| F-2974 | Horse | W Beringia |  | 5,9 | -20,9 | 4 |
| F-2967 | Horse | W Beringia |  | 5,5 | -21,1 | 4 |
| F-2970 | Horse | W Beringia |  | 8,9 | -20,7 | 4 |
| F-3058 | Horse | W Beringia |  | 6,9 | -22 | 4 |
| F-3059 | Horse | W Beringia |  | 8,9 | -20,8 | 4 |
| IK02-164 | Lion | E Beringia | >41100 | 11,0 | -19,5 | 1 |
| TIT12-07 | Lion | E Beringia | >48420 | 9,2 | -18,7 | 1 |
| IK97-1001 | Lion | E Beringia | 30520 ± 180 | 8,5 | -22,5 | 1 |
| IK98-436 | Lion | E Beringia | 35710 ± 11008 | 8,8 | -18,1 | 1 |
| IK01-112 | Lion | E Beringia | 40900 ± 11004 | 7,8 | -18,8 | 1 |
| F-2678/48 | Lion | W Beringia | >61000 | 12,6 | -19,9 | 4 |
| F-2678/118 | Lion | W Beringia | >61000 | 12,5 | -19,8 | 4 |
| F-2678/119 | Lion | W Beringia | >61000 | 12,5 | -19,9 | 4 |
| F-2678/120 | Lion | W Beringia | >61000 | 12,5 | -19,9 | 4 |
| F-2678/69 | Lion | W Beringia | >61000 | 11,4 | -19,2 | 4 |
| F-2450 | Lion | W Beringia | >61000 | 9,6 | -19 | 4 |
| F-2651 | Lion | W Beringia |  | 11,8 | -20,4 | 4 |
| F-2671 | Lion | W Beringia |  | 12 | -19,1 | 4 |
| F-278 | Lion | W Beringia |  | 12 | -20,2 | 4 |
| F-150 | Lion | W Beringia |  | 12,4 | -19,8 | 4 |
| IK99-070 | Mammoth | E Beringia | >48900 | 9,5 | -22,3 | 1 |
| IK01-132 | Mammoth | E Beringia | >49000 | 6,6 | -21,5 | 1 |
| IK01-079 | Mammoth | E Beringia | >49100 | 8,4 | -23,2 | 1 |
| T02-038 | Mammoth | E Beringia | >49100 | 6,6 | -22,4 | 1 |
| IK01-166 | Mammoth | E Beringia | >49200 | 6,6 | -20,9 | 1 |
| IK98-1312 | Mammoth | E Beringia | >50000 | 6,9 | -22,0 | 1 |
| IK98-1195 | Mammoth | E Beringia | >50100 | 6,1 | -21,1 | 1 |
| IK98-1012 | Mammoth | E Beringia | >50600 | 7,1 | -21,4 | 1 |
| IK98-1281 | Mammoth | E Beringia | >51000 | 7,2 | -21,4 | 1 |
| IK98-1274 | Mammoth | E Beringia | >51100 | 7,2 | -21,7 | 1 |
| IK99-130 | Mammoth | E Beringia | >51300 | 6,8 | -21,4 | 1 |
| IK98-1243 | Mammoth | E Beringia | >51500 | 6,8 | -21,7 | 1 |
| IK01-255 | Mammoth | E Beringia | >52000 | 7,7 | -22,0 | 1 |
| IK98-0759 | Mammoth | E Beringia | >52100 | 9,3 | -21,2 | 1 |
| IK01-274 | Mammoth | E Beringia | >52200 | 9,8 | -21,4 | 1 |
| IK02-042 | Mammoth | E Beringia | >53400 | 6,4 | -21,4 | 1 |
| IK01-132 | Mammoth | E Beringia | >54000 | 6,6 | -21,5 | 1 |
| IK01-082 | Mammoth | E Beringia | >54500 | 5,1 | -20,8 | 1 |
| IK98-0801 | Mammoth | E Beringia | >55100 | 7,9 | -20,8 | 1 |
| IK98-1195 | Mammoth | E Beringia | >55500 | 6,1 | -21,1 | 1 |
| IK98-1013 | Mammoth | E Beringia | >55500 | 7,4 | -21,8 | 1 |
| IK98-0339 | Mammoth | E Beringia | 24460 ± 130 | 7,6 | -21,5 | 1 |
| IK99-120b | Mammoth | E Beringia | 24850 ± 110 | 7,9 | -21,2 | 1 |
| IK01-040 | Mammoth | E Beringia | 28200 ± 200 | 7,6 | -21,2 | 1 |
| IK01-040 | Mammoth | E Beringia | 28220 ± 210 | 7,6 | -21,2 | 1 |
| IK98-0063 | Mammoth | E Beringia | 29100 ± 200 | 8,3 | -21,1 | 1 |
| IK99-495 | Mammoth | E Beringia | 29250 ± 200 | 8,0 | -21,2 | 1 |
| IK98-0063 | Mammoth | E Beringia | 29740 ± 190 | 8,3 | -21,1 | 1 |
| IK98-1275 | Mammoth | E Beringia | 30730 ± 200 | 7,1 | -21,8 | 1 |
| IK98-1102 | Mammoth | E Beringia | 40760 ± 830 | 7,8 | -21,4 | 1 |
| IK98-1102 | Mammoth | E Beringia | 45100 ± 1400 | 7,8 | -21,4 | 1 |
| IK98-0339 | Mammoth | E Beringia | 46770 ± 1710 | 7,6 | -21,5 | 1 |
| IK98-1013 | Mammoth | E Beringia | 46900 ± 1400 | 7,4 | -21,8 | 1 |
| IK01-250 | Mammoth | E Beringia | 47300 ± 1500 | 6,7 | -21,5 | 1 |
| IK01-250 | Mammoth | E Beringia | 47300 ± 1800 | 6,7 | -21,5 | 1 |
| IK98-0957 | Mammoth | E Beringia | 48760 ± 2200 | 8,1 | -20,7 | 1 |
| IK98-1033 | Mammoth | E Beringia | 49310 ± 19005 | 7,4 | -21,4 | 1 |
| IK01-255 | Mammoth | E Beringia | 49700 ± 2500 | 7,7 | -22,0 | 1 |
| IK98-0298 | Mammoth | E Beringia | 50400 ± 2700 | 7,8 | -22,1 | 1 |
| IK01-082 | Mammoth | E Beringia | 50800 ± 2400 | 5,1 | -20,8 | 1 |
| IK02-173 | Mammoth | E Beringia | 51800 ± 2700 | 6,4 | -21,3 | 1 |
| IK99-070 | Mammoth | E Beringia | 51900 ± 3200 | 9,5 | -22,3 | 1 |
| IK01-079 | Mammoth | E Beringia | 51900 ± 3200 | 8,4 | -23,2 | 1 |
| IK98-1243 | Mammoth | E Beringia | 53000 ± 3700 | 6,8 | -21,7 | 1 |
| IK98-0761 | Mammoth | E Beringia | 53400 ± 3900 | 7,7 | -20,8 | 1 |
| YU5.46 | Mammoth | E Beringia | 27098 | 9,4 | -21,0 | 9 |
| NMC6746 | Mammoth | E Beringia | 27745 | 9,7 | -21,0 | 9 |
| AM2446 | Mammoth | E Beringia | 30889 | 6,4 | -20,8 | 9 |
| YU3.256 | Mammoth | E Beringia | 33436 | 6,8 | -20,6 | 9 |
| YU3.133 | Mammoth | E Beringia | 33598 | 7,2 | -20,5 | 9 |
| YU3.136 | Mammoth | E Beringia | 33598 | 7,1 | -20,9 | 9 |
| NMC42135 | Mammoth | E Beringia | 34276 | 7,8 | -20,7 | 9 |
| YU52.36 | Mammoth | E Beringia | 34944 | 6,4 | -20,9 | 9 |
| YU133.18 | Mammoth | E Beringia | 36537 | 8,1 | -20,7 | 9 |
| YU5.130 | Mammoth | E Beringia | 36990 | 6,9 | -20,6 | 9 |
| YU130.2 | Mammoth | E Beringia | 41653 | 6,6 | -20,5 | 9 |
| NMC42292 | Mammoth | E Beringia | 41782 | 7,9 | -20,6 | 9 |
| YU137.3 | Mammoth | E Beringia | 43724 | 7,3 | -20,5 | 9 |
| AM523 | Mammoth | E Beringia | 47030 | 4,4 | -20,8 | 9 |
| YU3.19 | Mammoth | E Beringia | 48491 | 5,7 | -20,6 | 9 |
| NMC49928 | Mammoth | E Beringia | 49485 | 6,9 | -20,3 | 9 |
| YU3.135 | Mammoth | E Beringia | 49709 | 6,4 | -20,6 | 9 |
| YU136.9 | Mammoth | E Beringia | 49985 | 7,5 | -20,8 | 9 |
| NMC49927 | Mammoth | E Beringia | 50002 | 8,7 | -20,4 | 9 |
| NMC49562 | Mammoth | E Beringia | 50867 | 5,8 | -21,3 | 9 |
| YU5.69 | Mammoth | E Beringia | 51282 | 9,0 | -20,7 | 9 |
| YU57.1 | Mammoth | E Beringia | 51652 | 9,3 | -21,2 | 9 |
| AM1114 | Mammoth | E Beringia | 59583 | 5,0 | -20,5 | 9 |
| 94200 | Mammoth | W Beringia | 30000 | 9,1 | -22,4 | 8 |
| 95100 | Mammoth | W Beringia | 50000 | 8,8 | -22,7 | 8 |
| 16/2 | Mammoth | W Beringia | *40000* | 6,0 | -21,9 | 2 |
| 19/52 | Mammoth | W Beringia | 30100 ± 550 | 9,1 | -22,1 | 2 |
| 19/58 | Mammoth | W Beringia | 40200 ± 1800 | 11,2 | -21,6 | 2 |
| M, primigenius 23 | Mammoth | W Beringia | 34600 | 9,1 | -22,2 | 3 |
| M, primigenius 3 | Mammoth | W Beringia | 13100 ± 500 | 5,6 | -22,7 | 3 |
| M, primigenius 7 | Mammoth | W Beringia | 20800 ± 600 | 8,3 | -21,7 | 3 |
| M, primigenius 9 | Mammoth | W Beringia | 24300 ± 200 | 7,3 | -22,3 | 3 |
| M, primigenius 10 | Mammoth | W Beringia | 25900 ± 600 | 8,9 | -22,5 | 3 |
| M, primigenius 11 | Mammoth | W Beringia | 27400 ± 800 | 9,1 | -21,9 | 3 |
| M. primigenius 12 | Mammoth | W Beringia | 28000 ± 180 | 9,0 | -21,6 | 3 |
| M, primigenius 13 | Mammoth | W Beringia | 30200 ± 400 | 9,1 | -22,1 | 3 |
| M, primigenius 14 | Mammoth | W Beringia | 31500 ± 650 | 8,9 | -21,7 | 3 |
| M. primigenius A | Mammoth | W Beringia | 32500 ± 500 | 9,2 | -22,6 | 3 |
| M, primigenius 15 | Mammoth | W Beringia | 34000 ± 500 | 8,6 | -22,0 | 3 |
| M. primigenius 16 | Mammoth | W Beringia | 37800 ± 900 | 8,8 | -21,9 | 3 |
| M. primigenius 17 | Mammoth | W Beringia | 39600 ± 1000 | 9,4 | -22,5 | 3 |
| M. primigenius 18 | Mammoth | W Beringia | 40200 ± 900 | 9,6 | -22,3 | 3 |
| M. primigenius 19 | Mammoth | W Beringia | 43600 ± 1000 | 8,7 | -23,2 | 3 |
| M. primigenius 20 | Mammoth | W Beringia | 48000 ± 2000 | 7,6 | -21,7 | 3 |
| M. primigenius 21 | Mammoth | W Beringia | 50650 ± 1820 | 9,3 | -22,3 | 3 |
| No number | Mammoth | W Beringia |  | 12 | -20,9 | 4 |
| F-3044 | Mammoth | W Beringia |  | 9,5 | -21 | 4 |
| F-1885 | Mammoth | W Beringia |  | 9,6 | -21,7 | 4 |
| F-1915 | Mammoth | W Beringia |  | 9,1 | -21,6 | 4 |
| F-3040 | Mammoth | W Beringia |  | 9,7 | -21,8 | 4 |
| F-3030 | Mammoth | W Beringia |  | 9,9 | -21,5 | 4 |
| F-2403 | Mammoth | W Beringia |  | 10 | -22 | 4 |
| F-3043 | Mammoth | W Beringia |  | 7,9 | -20,7 | 4 |
| F-2402 | Mammoth | W Beringia |  | 7,7 | -21,3 | 4 |
| F-3045 | Mammoth | W Beringia |  | 10,2 | -22,1 | 4 |
| F-2757/35 | Mammoth | W Beringia |  | 11 | -22,3 | 4 |
| F-3034 | Mammoth | W Beringia |  | 10,8 | -21,4 | 4 |
| F-2398 | Mammoth | W Beringia |  | 8,7 | -21,4 | 4 |
| F-768 | Mammoth | W Beringia |  | 9,9 | -21,4 | 4 |
| F-300/1 | Mammoth | W Beringia |  | 10,8 | -21,4 | 4 |
| F-604 | Mammoth | W Beringia |  | 7,7 | -21,5 | 4 |
| F-600 | Mammoth | W Beringia |  | 9,2 | -21,8 | 4 |
| F-2644 | Mammoth | W Beringia |  | 9,3 | -22,3 | 4 |
| 2005/945 | Mammoth | W Beringia | 24008 | 9,1 | -20,8 | 9 |
| 2005/915 | Mammoth | W Beringia | 32312 | 10,0 | -21,9 | 9 |
| 2000/174 | Mammoth | W Beringia | 32640 | 10,7 | -21,7 | 9 |
| 2005/900 | Mammoth | W Beringia | 33169 | 9,7 | -21,9 | 9 |
| 2005/917 | Mammoth | W Beringia | 40246 | 8,7 | -21,4 | 9 |
| 2005/897 | Mammoth | W Beringia | 43920 | 8,9 | -21,7 | 9 |
| 2005/907 | Mammoth | W Beringia | 44649 | 8,1 | -22,1 | 9 |
| 2006/001 | Mammoth | W Beringia | 44800 | 8,1 | -21,7 | 9 |
| 2001/412 | Mammoth | W Beringia | 47755 | 10,5 | -21,8 | 9 |
| 2002/473 | Mammoth | W Beringia | 51013 | 11,4 | -22,0 | 9 |
| SYU3 | Mammoth | W Beringia | 51652 | 10,9 | -22,6 | 9 |
| 2002/472 | Mammoth | W Beringia | 52803 | 10,0 | -22,0 | 9 |
| 2005/999 | Mammoth | W Beringia | 54260 | 7,5 | -22,0 | 9 |
| AMNH F:AM 142439 | Reindeer | E Beringia | 40700 | 4,7 | -19,4 | 7 |
| AMNH F:AM 142442 | Reindeer | E Beringia | 40700 | 2,1 | -19,3 | 7 |
| AMNH F:AM 142445 | Reindeer | E Beringia | 41100 | 3,7 | -19,7 | 7 |
| AMNH F:AM 142447 | Reindeer | E Beringia | 45200 | 1,7 | -19,2 | 7 |
| AMNH F:AM 142446 | Reindeer | E Beringia | 29640 ± 370 | 3,1 | -19,3 | 7 |
| AMNH FM 142446 | Reindeer | E Beringia | 29640 ± 370 | 3,1 | -19,3 | 7 |
| AMNH FM 142444 | Reindeer | E Beringia | >40673 | 4,7 | -19,4 | 7 |
| AMNH FM 142443 | Reindeer | E Beringia | >40707 | 2,1 | -19,3 | 7 |
| AMNH FM 142445 | Reindeer | E Beringia | >41100 | 3,7 | -19,7 | 7 |
| AMNH FM 142447 | Reindeer | E Beringia | >45200 | 1,7 | -19,9 | 7 |
| M-98-064 | Reindeer | E Beringia | 24750 ± 110 | 6,2 | -19,2 | 1 |
| IK98-0690 | Reindeer | E Beringia | 24940 ± 110 | 6,3 | -17,9 | 1 |
| IK98-0311 | Reindeer | E Beringia | 27420 ± 190 | 3,7 | -18,4 | 1 |
| IK98-0833 | Reindeer | E Beringia | 28760 ± 160 | 7,0 | -18,5 | 1 |
| IK98-0079 | Reindeer | E Beringia | 28930 ± 170 | 3,3 | -18,3 | 1 |
| IK98-1207 | Reindeer | E Beringia | 30050 ± 210 | 3,8 | -18,2 | 1 |
| IK99-171 | Reindeer | E Beringia | 30270 ± 200 | 3,9 | -19,0 | 1 |
| IK99-570 | Reindeer | E Beringia | 30820 ± 200 | 2,3 | -17,7 | 1 |
| IK99-409 | Reindeer | E Beringia | 31830 ± 330 | 4,0 | -18,2 | 1 |
| IK98-0983 | Reindeer | E Beringia | 31920 ± 240 | 2,6 | -18,6 | 1 |
| IK98-0479 | Reindeer | E Beringia | 32570 ± 250 | 1,6 | -17,7 | 1 |
| IK99-543 | Reindeer | E Beringia | 37570 ± 580 | 3,8 | -18,1 | 1 |
| IK99-066 | Reindeer | E Beringia | 38830 ± 540 | 5,4 | -18,1 | 1 |
| IK98-0350 | Reindeer | E Beringia | 40840 ± 950 | 1,0 | -18,0 | 1 |
| IK98-0450 | Reindeer | E Beringia | 41100 ± 1100 | 2,4 | -19,4 | 1 |
| IK99-764 | Reindeer | E Beringia | 42040 ± 800 | 1,8 | -18,5 | 1 |
| IK98-0478 | Reindeer | E Beringia | 42500 ± 1200 | 3,0 | -17,3 | 1 |
| IK98-0041 | Reindeer | E Beringia | 44200 ± 1000 | 1,2 | -17,8 | 1 |
| IK98-0687 | Reindeer | E Beringia | 44300 ± 1100 | 1,9 | -17,2 | 1 |
| IK98-1051 | Reindeer | E Beringia | 45500 ± 1200 | 3,9 | -18,7 | 1 |
| IK98-1108 | Reindeer | E Beringia | 46300 ± 1400 | 3,3 | -18,1 | 1 |
| IK98-0310 | Reindeer | E Beringia | 46500 ± 1600 | 5,0 | -19,7 | 1 |
| IK98-0153 | Reindeer | E Beringia | 48000 ± 2600 | 1,7 | -18,1 | 1 |
| IK99-286 | Reindeer | E Beringia | 48300 ± 1700 | 2,0 | -17,5 | 1 |
| IK98-0031 | Reindeer | E Beringia | 48300 ± 2100 | 8,7 | -19,9 | 1 |
| IK99-199 | Reindeer | E Beringia | 52000 ± 2700 | 1,8 | -17,6 | 1 |
| IK98-0573 | Reindeer | E Beringia | 52600 ± 3500 | 3,1 | -19,7 | 1 |
| IK98-1230 | Reindeer | E Beringia | 53300 ± 3800 | 1,5 | -17,8 | 1 |
| IK98-1227 | Reindeer | E Beringia | >46900 | 1,7 | -18,5 | 1 |
| IK98-0351 | Reindeer | E Beringia | >47200 | 4,3 | -18,9 | 1 |
| IK98-1228 | Reindeer | E Beringia | >49900 | 4,3 | -18,0 | 1 |
| IK98-1158 | Reindeer | E Beringia | >49900 | 0,1 | -17,5 | 1 |
| IK99-585 | Reindeer | E Beringia | >51200 | 2,3 | -17,2 | 1 |
| IK99-247 | Reindeer | E Beringia | >51700 | 4,6 | -18,5 | 1 |
| IK99-740 | Reindeer | E Beringia | >52800 | 2,9 | -17,0 | 1 |
| F-3027 | Reindeer | W Beringia |  | 4,5 | -17,9 | 4 |
| F-3027 | Reindeer | W Beringia |  | 5,4 | -18,9 | 4 |
| F-3025 | Reindeer | W Beringia |  | 3,3 | -19,7 | 4 |
| F-3025 | Reindeer | W Beringia |  | 5,7 | -20,3 | 4 |
| F-3029 | Reindeer | W Beringia |  | 7 | -19,5 | 4 |
| F-3024 | Reindeer | W Beringia |  | 7,8 | -20,7 | 4 |
| no number | Reindeer | W Beringia |  | 4,7 | -19,4 | 4 |
| F-2373 | Reindeer | W Beringia |  | 6,9 | -19,4 | 4 |
| F-3066 | Reindeer | W Beringia |  | 7,9 | -20,5 | 4 |
| F-3065 | Reindeer | W Beringia |  | 1,7 | -19,2 | 4 |
| F-3065 | Reindeer | W Beringia |  | 1,9 | -19,7 | 4 |
| AMNH F:AM 116840 | Scimitar Cat | E Beringia | 40500 | 9,7 | -20,1 | 10 |
| AMNH F:AM 128069 | Scimitar Cat | E Beringia | 41000 | 5,5 | -18,3 | 10 |
| AMNH F:AM 142497 | Scimitar Cat | E Beringia | 42500 | 8,2 | -19,0 | 10 |
| AMNH F:AM 142496 | Scimitar Cat | E Beringia | 43300 | 7,5 | -19,0 | 10 |
| AMNH F:AM 142493 | Scimitar Cat | E Beringia | 44200 | 5,9 | -19,4 | 10 |
| AMNH F:AM 142490 | Scimitar Cat | E Beringia | 36200±1300 | 6,6 | -20,7 | 10 |
| AMNH F:AM 142494 | Scimitar Cat | E Beringia | 36320±1270 | 9,2 | -18,9 | 10 |
| AMNH F:AM 95567 | Scimitar Cat | E Beringia | 41000±2300 | 8,0 | -19,1 | 10 |
| AMNH F:AM 30770i | Scimitar Cat | E Beringia | 41900±2600 | 9,2 | -19,2 | 10 |
| AMNH F:AM 142495 | Scimitar Cat | E Beringia | 43400±3100 | 9,0 | -19,7 | 10 |
| AMNH F:AM 142492 | Scimitar Cat | E Beringia | 47710±5270 | 8,8 | -19,3 | 10 |
| CMN 49874 | Short-faced bear | E Beringia | 26720 | 10,2 | -17,9 | 5 |
| AMNH F:AM 30494 | Short-faced bear | E Beringia | 25496±224 | 7,7 | -17,8 | 10 |
| AMNH A-37-10 | Short-faced bear | E Beringia | 27511±279 | 9,5 | -18,3 | 10 |
| AMNH 99209 | Short-faced bear | E Beringia | 39565±1126 | 8,5 | -18,1 | 10 |
| NMC 7438 | Short-faced bear | E Beringia | 26040 ± 270 | 10,3 | -18,5 | 6 |
| NMC 37577 | Short-faced bear | E Beringia | 29600 ± 1200 | 9,7 | -19,0 | 6 |
| AMNH F:AM 67163 | Wolf | E Beringia | 31800 | 6,6 | -18,8 | 7; 10 |
| AMNH F:AM 67228 | Wolf | E Beringia | 32100 | 7,0 | -19,4 | 7; 10 |
| AMNH F:AM 30440 | Wolf | E Beringia | 38000 | 6,4 | -18,9 | 7; 10 |
| AMNH F:AM 67235 | Wolf | E Beringia | 38000 | 5,8 | -19,8 | 7; 10 |
| AMNH F:AM 70946 | Wolf | E Beringia | 38000 | 5,4 | -20,0 | 7; 10 |
| AMNH F:AM 142410 | Wolf | E Beringia | 45400 | 6,2 | -19,4 | 7; 10 |
| AMNH F:AM 142409 | Wolf | E Beringia | 48130 | 8,6 | -19,4 | 7; 10 |
| AMNH F:AM 67170 | Wolf | E Beringia | 27620±580 | 7,3 | -18,3 | 7; 10 |
| AMNH F:AM 30431 | Wolf | E Beringia | 28500±300 | 8,6 | -19,1 | 7; 10 |
| AMNH F:AM 67248 | Wolf | E Beringia | 29800±400 | 6,2 | -19,7 | 7; 10 |
| AMNH F:AM 67168 | Wolf | E Beringia | 31200±450 | 9,2 | -19,0 | 7; 10 |
| CMN 42388 | Wolf | E Beringia | 33900±1700 | 8,5 | -18,5 | 7; 10 |
| AMNH F:AM 67184 | Wolf | E Beringia | 34600±700 | 6,2 | -20,2 | 7; 10 |
| AMNH F:AM 67159 | Wolf | E Beringia | 35200±2300 | 8,1 | -19,4 | 7; 10 |
| AMNH F:AM 70958 | Wolf | E Beringia | 37700±2600 | 7,5 | -19,1 | 7; 10 |
| AMNH F:AM 70945 | Wolf | E Beringia | 37733±2633 | 6,7 | -18,8 | 7; 10 |
| AMNH F:AM 67202 | Wolf | E Beringia | 38000±2700 | 6,8 | -19,2 | 7; 10 |
| AMNH F:AM 67243 | Wolf | E Beringia | 38500±1100 | 6,2 | -20,1 | 7; 10 |
| CMN 17311 | Wolf | E Beringia | 38790±540 | 6,1 | -19,1 | 7; 10 |
| AMNH F:AM 67197 | Wolf | E Beringia | 39300±1230 | 5,7 | -20,3 | 7; 10 |
| AMNH F:AM 67208 | Wolf | E Beringia | 41040±1530 | 9,7 | -19,2 | 7; 10 |
| AMNH F:AM 30438 | Wolf | E Beringia | 45500±2700 | 6,4 | -19,4 | 7; 10 |
| AMNH F:AM 67167 | Wolf | E Beringia | 45800±2800 | 9,2 | -19,0 | 7; 10 |
| IK08-096 | Wolf | E Beringia | >43500 | 8,9 | -20,0 | 1 |
| F-945 | Wolf | W Beringia |  | 10,7 | -21,1 | 4 |
| F-2672 | Wolf | W Beringia |  | 13,3 | -20 | 4 |
| F-933 | Wolf | W Beringia |  | 11,2 | -19,6 | 4 |
| No number | Wolf | W Beringia |  | 9,7 | -19,9 | 4 |
| F-3055 | Wolf | W Beringia |  | 12,9 | -19,1 | 4 |
| F-3056 | Wolf | W Beringia |  | 9,9 | -20,9 | 4 |

**References**

1. Mann, D. H., Groves, P., Kunz, M. L., Reanier, R. E. & Gaglioti, B. V. Ice-age megafauna in Arctic Alaska: extinction, invasion, survival. *Quat. Sci. Rev.* **70,** 91–108 (2013).

2. Iacumin, P., Nikolaev, V. & Ramigni, M. C and N stable isotope measurements on Eurasian fossil mammals, 40 000 to 10 000 years BP: Herbivore physiologies and palaeoenvironmental reconstruction. *Palaeogeogr. Palaeoclimatol. Palaeoecol.* **163,** 33–47 (2000).

3. Iacumin, P., Di Matteo, A., Nikolaev, V. & Kuznetsova, T. V. Climate information from C, N and O stable isotope analyses of mammoth bones from northern Siberia. *Quat. Int.* **212,** 206–212 (2010).

4. Kirillova, I. V *et al.* On the discovery of a cave lion from the Malyi Anyui River (Chukotka, Russia). *Quat. Sci. Rev.* **117,** 135–151 (2015).

5. Barnes, I., Matheus, P., Shapiro, B., Jensen, D. & Cooper, A. Dynamics of Pleistocene Population Extinctions in Beringian Brown Bears. *Science (80-. ).* **295,** 2267–2270 (2002).

6. Matheus, P. Diet and Co-ecology of Pleistocene Short-Faced Bears and Brown Bears in Eastern Beringia. *Quat. Res.* **44,** 447–453 (1995).

7. Leonard, J. A. *et al.* Megafaunal Extinctions and the Disappearance of a Specialized Wolf Ecomorph. *Curr. Biol.* **17,** 1146–1150 (2007).

8. Bocherens, H., Pacaud, G., Lazarev, P. A. & Mariotti, A. Stable isotope abundances (13C, 15N) in collagen and soft tissues from Pleistocene mammals from Yakutia: Implications for the palaeobiology of the Mammoth Steppe. *Palaeogeogr. Palaeoclimatol. Palaeoecol.* **126,** 31–44 (1996).

9. Szpak, P. *et al.* Regional differences in bone collagen δ13C and δ15N of Pleistocene mammoths: Implications for paleoecology of the mammoth steppe. *Palaeogeogr. Palaeoclimatol. Palaeoecol.* **286,** 88–96 (2010).

10. Fox-Dobbs, K., Leonard, J. A. & Koch, P. L. Pleistocene megafauna from eastern Beringia: Paleoecological and paleoenvironmental interpretations of stable carbon and nitrogen isotope and radiocarbon records. *Palaeogeogr. Palaeoclimatol. Palaeoecol.* **261,** 30–46 (2008).

Supplementary Table 6. Mean and standard deviation (SD) of *δ*^15^N and *δ*^13^C, and differences between the means of the population of every species of Eastern (E) and Western (W) Beringia.

| **Species** | **N** | **Mean *δ*^15^N** | **Mean *δ*^13^C** | **SD *δ*^15^N** | **SD *δ*^13^C** | **Δ *δ*^15^N** | **Δ *δ*^13^C** |
| --- | --- | --- | --- | --- | --- | --- | --- |
| Bison - E | 59 | 4.7 | -20.0 | 1.20 | 0.40 | 2.7 | -1.1 |
| Bison - W | 15 | 7.4 | -21.1 | 1.10 | 0.45 |  |  |
| Brown bear - E | 11 | 6.4 | -19.5 | 1.55 | 0.40 | 4.6 | -0.5 |
| Brown bear -W | 9 | 11 | -20.0 | 1.43 | 0.70 |  |  |
| Horse - E | 77 | 5.4 | -21.1 | 2.03 | 0.38 | 1.0 | 0.0 |
| Horse - W | 21 | 6.3 | -21.1 | 1.47 | 0.37 |  |  |
| Lion - E | 5 | 9.1 | -19.5 | 1.19 | 1.74 | 2.9 | -0.2 |
| Lion - W | 10 | 11.9 | -19.7 | 0.91 | 0.47 |  |  |
| Mammoth - E | 68 | 7.3 | -21.3 | 1.15 | 0.61 | 1.9 | -0.6 |
| Mammoth - W | 53 | 9.2 | -21.9 | 1.24 | 0.50 |  |  |
| Reindeer - E | 45 | 3.3 | -18.5 | 1.71 | 0.83 | 1.9 | -1.0 |
| Reindeer - W | 11 | 5.2 | -19.6 | 2.19 | 0.78 |  |  |
| Scimitar Cat - E | 11 | 8.0 | -19.3 | 1.42 | 0.64 |  |  |
| Short-faced bear - E | 6 | 9.3 | -18.3 | 1.02 | 0.43 |  |  |
| Wolf - E | 24 | 7.2 | -19.3 | 1.29 | 0.53 | 4.1 | -0.8 |
| Wolf - W | 6 | 11.3 | -20.1 | 1.51 | 0.77 |  |  |
| Horse & Bison - E | 136 | 5.1 | -20.6 | 1.74 | 0.65 | 1.7 | -0.5 |
| Horse & Bison - W | 36 | 6.8 | -21.1 | 1.41 | 0.40 |  |  |
